# Rapid detection of IDH mutations in gliomas by intraoperative mass spectrometry

**DOI:** 10.1073/pnas.2318843121

**Published:** 2024-05-28

**Authors:** Wei Hua, Wenpeng Zhang, Hannah Brown, Junhan Wu, Xinqi Fang, Mahdiyeh Shahi, Rong Chen, Haoyue Zhang, Bin Jiao, Nan Wang, Hao Xu, Minjie Fu, Xiaowen Wang, Jinsen Zhang, Xin Zhang, Qijun Wang, Wei Zhu, Dan Ye, Diogo Moniz Garcia, Kaisorn Chaichana, R. Graham Cooks, Zheng Ouyang, Ying Mao, Alfredo Quinones-Hinojosa

**Affiliations:** ^a^Department of Neurosurgery, Huashan Hospital, Fudan University, Shanghai 200040, China; ^b^National Center for Neurological Disorders, Shanghai 200040, China; ^c^Shanghai Key Laboratory of Brain Function Restoration and Neural Regeneration, Shanghai 200040, China; ^d^Neurosurgical Institute of Fudan University, Shanghai 200040, China; ^e^Shanghai Clinical Medical Center of Neurosurgery, Shanghai 200040, China; ^f^State Key Laboratory of Precision Measurement Technology and Instruments, Department of Precision Instrument, Tsinghua University, Beijing 100084, China; ^g^Department of Chemistry, Purdue University, West Lafayette, IN 47907; ^h^PurSpecTechnologies, Beijing 100084, China; ^i^The Molecular and Cell Biology Lab, Institute of Biomedical Sciences, Shanghai Medical College, Fudan University, Shanghai 200232, China; ^j^Department of Neurosurgery, Mayo Clinic, Jacksonville, FL 32224

**Keywords:** intraoperative analysis, tandem mass spectrometry, 2-hydroxyglutarate, molecular diagnostics, oncometabolite

## Abstract

The development and performance of two mass spectrometry (MS) workflows for the intraoperative diagnosis of isocitrate dehydrogenase (IDH) mutations in glioma is implemented by independent teams at Mayo Clinic, Jacksonville, and Huashan Hospital, Shanghai. The infiltrative nature of gliomas makes rapid diagnosis necessary to guide the extent of surgical resection of central nervous system (CNS) tumors. The combination of tissue biopsy and MS analysis used here satisfies this requirement. The key feature of both described methods is the use of tandem MS to measure the oncometabolite 2-hydroxyglutarate (2HG) relative to endogenous glutamate (Glu) to characterize the presence of mutant tumor. The experiments i) provide IDH mutation status for individual patients and ii) demonstrate a strong correlation of 2HG signals with tumor infiltration. The measured ratio of 2HG to Glu correlates with IDH-mutant (IDH-mut) glioma (*P* < 0.0001) in the tumor core data of both teams. Despite using different ionization methods and different mass spectrometers, comparable performance in determining IDH mutations from core tumor biopsies was achieved with sensitivities, specificities, and accuracies all at 100%. None of the 31 patients at Mayo Clinic or the 74 patients at Huashan Hospital were misclassified when analyzing tumor core biopsies. Robustness of the methodology was evaluated by postoperative re-examination of samples. Both teams noted the presence of high concentrations of 2HG at surgical margins, supporting future use of intraoperative MS to monitor for clean surgical margins. The power of MS diagnostics is shown in resolving contradictory clinical features, e.g., in distinguishing gliosis from IDH-mut glioma.

## Significance

With the arrival of genomics, molecular diagnostics has grown in importance as a complement to microscopic observations. In brain cancer, some gliomas have mutations in an enzyme associated with the isocitrate biochemical pathway which result in the accumulation of a distinctive metabolite, 2-hydroxyglutarate (2HG). Measurement of brain tissue during surgery using a mass spectrometer (MS) provides information on isocitrate dehydrogenase (IDH)-mutation status not available prior to or during surgery. The data suggest that MS could be used to identify IDH-mut tissue margins at locations of interest selected by the surgeon intraoperatively and rapidly within 2 min. Thus, this molecular measurement should assist the surgeon in achieving optimal surgical resection of infiltrated tissue in those cases where the tumor core is IDH-mutant positive.

Molecular markers are critical for the accurate diagnosis, prognosis, and for treatment of glioma, a prevalent form of brain cancer with poor 5-y survival rates (1, 2). These molecular features are of growing importance for patient prognosis and clinician decision-making, both intraoperatively and postoperatively, and there is an increasing desire to tailor treatment regimens to the molecular features of a patient’s specific tumor. The genetic subtyping of cancers, including gliomas, has improved the ability of surgeons to predict patient prognosis—specifically response to treatments and survival—relative to factors such as cellular morphology or age (3–5). Personalized medicine approaches emphasize the implementation of treatment protocols that are mostly likely to be effective in treating a patient’s specific tumor and so maximize survival benefit (6). To take advantage of the “window of opportunity” at the time of surgery, tailoring treatment to the molecular features of a tumor requires the ability to assess molecular features at the time of surgery. However, the principal source of intraoperative diagnostic information remains the microscopic review of tissue sections which is used only to confirm the initial diagnosis through a frozen section (7–10); it does not provide molecular or genetic information (11, 12).

**Fig. 1.**
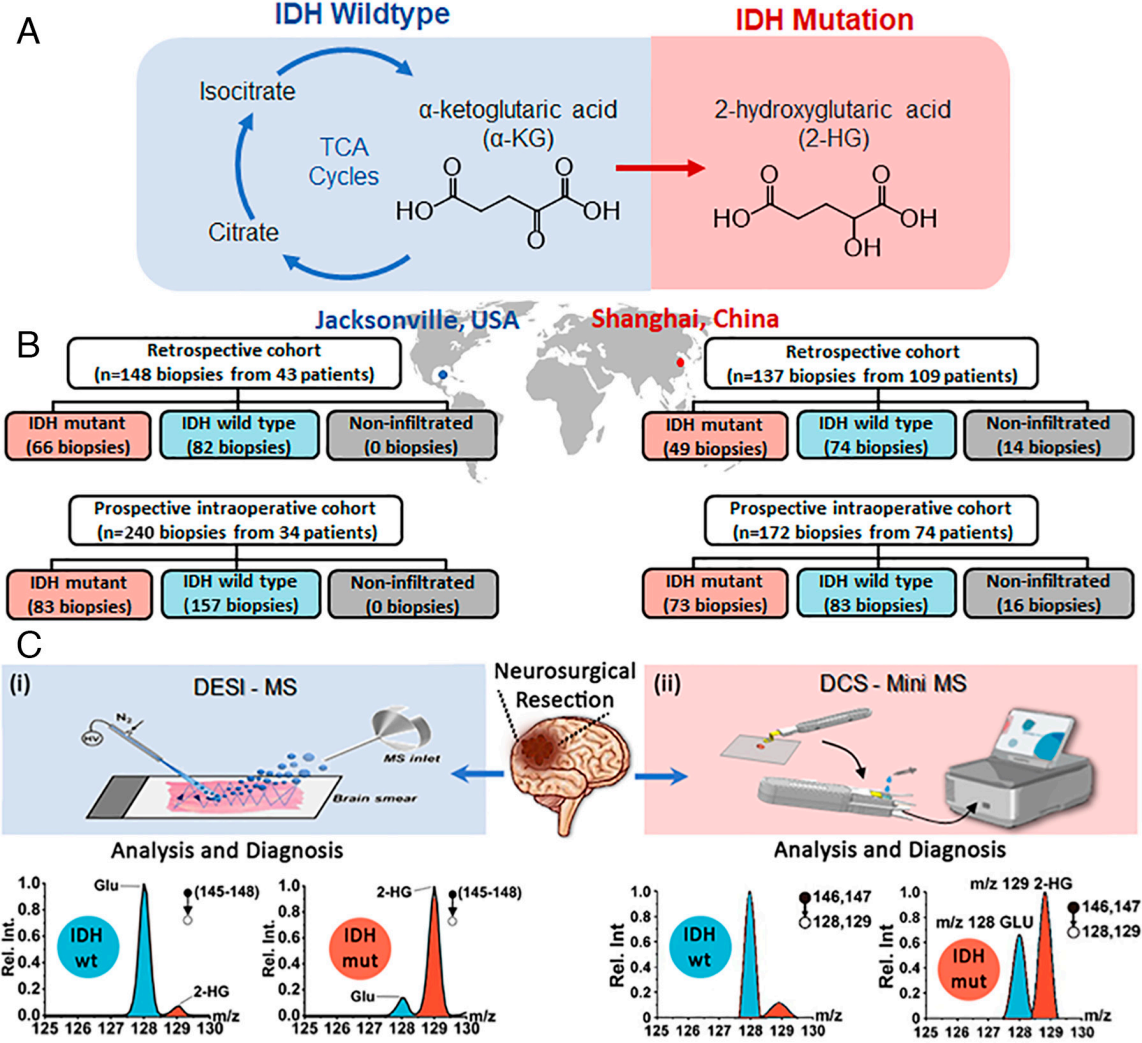
(*A*) IDH-mutant gliomas harbor significant metabolic aberrations. Specifically, α-ketoglutaric acid is reduced to 2HG which accumulates in IDH-mutant tumors. (*B*) Data on training (retrospective) and validation (prospective) cohorts in Mayo and Huashan studies. (*C*) Workflow of rapid metabolic analysis of gliomas by MS/MS measurement of 2HG (*m/z* 129) in IDH-mut gliomas using Glu (*m/z* 128) as reference. Typical results for (*i*) Mayo/Purdue study with DESI and benchtop ion trap MS and (*ii*) Huashan/Tsinghua study with direct capillary spray and miniature MS.

Isocitrate dehydrogenase (IDH) mutations alter enzymatic pathways that catalyze the conversion of isocitrate into α-ketoglutaric acid and confer neomorphic gain-of-function enzymatic properties, specifically the nicotinamide adenine dinucleotide phosphate (NADPH)-dependent reduction of α-ketoglutaric acid to the (normally trace) metabolite 2-hydroxyglutarate (2HG), Fig. 1*A* (13–16). Consequently, 2HG accumulates dramatically in IDH-mutant (IDH-mut) tumor cells, reaching concentrations of up to 35 μmol per gram of tissue (15) while remaining extremely low in adjacent paratumor brain tissue. Knowledge of IDH mutations is important for diagnosis, prognosis, and treatment of glioma patients (both intraoperatively and postoperatively) (17, 18). IDH mutations play an essential role in the determination of glioma classification which, until recently, was based solely on morphological features. However, in 2016, the World Health Organization (WHO) included specific molecular features, among them the IDH genotype (19) and expanded the use of molecular features in the 2021 WHO Classification of Tumors of the Central Nervous System (2). Glioma diagnosis now includes IDH-wild type (IDH-wt) and IDH-mut categories in addition to grade and glioma type (e.g., astrocytoma, oligodendroglioma, glioblastoma) in the standard-of-care diagnostic approach (2). Furthermore, knowledge of IDH mutations has prognostic value in that patients with IDH-mut tumors have longer survival rates than those with IDH-wt tumors (20–22).

While our understanding of the molecular diversity of gliomas has increased greatly, the available treatments for glioma patients remain mostly unchanged. Surgery and its extent of resection are still the most important prognostic modulators in gliomas, with the distinction between infiltrated and noninfiltrated tissues being a key question, particularly at the penumbra (21, 22). This infiltrative nature highlights the need for more accurate diagnostic and imaging modalities to guide surgical resection and minimize iatrogenic deficits (23, 24). The methods currently available for the estimation of tumor infiltration in surgical biopsies are discussed below; their limitations are such that this approach is not routinely performed (11, 12, 25). Furthermore, rapid assessment of tumor infiltration in biopsied tissue near surgical resection margins is also not currently routine as it can be time-consuming and only gives a preliminary assessment through a frozen section. A rapid and accurate molecular measurement (e.g., the detection of 2HG as an indicator of the presence of tumor cells in IDH-mut gliomas) could help improve resection and patient management.

Despite knowledge of the diagnostic value of molecular features, the intraoperative assessment of molecular features in brain tissue biopsies, including IDH mutations, is rarely performed because accurate methods are just now being reported. Currently, IDH mutations can be identified, postoperatively, by immunohistochemical staining or by PCR sequencing (26, 27). Medical imaging methods, including MRI and computed tomography (CT) inform surgical approach and extent of resection but do not provide direct information on molecular features (28) although preliminary MRI indications show that preoperative imaging can provide limited molecular assessment (29). Magnetic resonance spectroscopy (MRS), an emerging method capable of detecting unique signals from molecules of interest, holds promise as a noninvasive method for the detection and quantification of clinically relevant biomarkers, although widespread implementation is yet to be realized (30). Similar comments apply to the use of intraoperative Raman spectroscopy (31). Consequently, the diagnostic consultation is incomplete, and at times inconclusive, since the accurate diagnosis of brain tumors relies heavily on the assessment of molecular features that are currently only performed postoperatively, with results available several days after surgery.

In order for an intraoperative molecular technique to be widely used, it must provide reliable molecular data rapidly (within a few minutes) from minimally processed tissue biopsies. Mass spectrometry (MS) is a highly sensitive and powerful analytical technique capable of detecting and quantifying molecules in complex samples (32, 33). However, MS is generally not applicable to the identification and quantitation of individual compounds in complex samples like tissue unless augmented by chromatographic separation or by tandem mass spectrometry (MS/MS). MS experiments performed in open air are valuable diagnostics for various diseases (34). Intraoperative use of MS has historically faced barriers to implementation due to the time required for sample preparation and for online chromatographic separation. Newer extractive MS methods like the MasSpec Pen (35), desorption electrospray ionization (DESI) (36), and direct capillary spray (DCS)(37), when combined with MS/MS, overcome these barriers. The first of these methods allows intrasurgical measurements but has not yet been used in brain cancer while the latter two methods are used with the two workflows in the present study to provide relative concentrations of 2HG in tissue. Independent workflows were performed by researchers from Purdue University and Tsinghua University at Mayo Clinic, Jacksonville, Florida, United States of America, and Huashan Hospital, Shanghai, China, respectively. Two categories of information are reported: i) IDH mutation status and ii) information on the correlation of 2HG with tumor infiltration.

## Results

Fig. 1*B* summarizes patient numbers used in the training cohort and validation cohort in Purdue University and Tsinghua University studies. Note that the Purdue University training study utilized some previously reported data as well as data new to this study (see *SI Appendix*, Tables S1 and S2 for details). Two different ionization methods, DESI and DCS, were used to generate ions from fresh, unmodified tissue biopsies intraoperatively. The same methods were used for the banked samples which were examined after thawing. Both methods have previously been used for intraoperative brain tissue diagnostics (36, 38–40). The two methods are summarized in Fig. 1*C*, together with typical results. Note that both mass spectrometers used were ion traps although one was a miniature instrument and the other a modified benchtop instrument (39, 41).

Prior studies (2015 to 2019) at Methodist Hospital, Indianapolis led to the selection of the ratiometric MS/MS methodology employed in this study (36, 38–40, 42). The metabolite signals recorded in single-stage mass spectra overlap those for other molecules, so tandem MS (MS/MS) was used to provide the needed molecular selectivity and measurement accuracy. Glutamate (Glu), an abundant molecule in the brain not associated with IDH mutations and whose concentration varies inversely with 2HG (43, 44), was used as an endogenous reference in the MS/MS measurement. The ratio of the signals detected in the simultaneous analysis of 2HG and Glu is used to assess IDH mutation status. In all cases, data were recorded at unit resolution, and to improve the accuracy of the determinations, the interference caused by isotopes was automatically calculated and subtracted from the measured ion ratios (*SI Appendix*, Fig. S1). Despite using different ionization methods and mass spectrometers, both methods produced similar mass spectra and used similar ratiometric approaches to determine IDH mutation status. It should be noted that this method cannot differentiate IDH-wt gliomas from normal brain tissue.

Building on successes of previous studies (36, 38–40, 45), both teams set out to develop intraoperative MS methods for the determination of IDH mutation status using different ionization methods and different MS systems (benchtop and miniature). The primary information sought was the quality of the correlation of 2HG with IDH-mut tumor cores. A secondary aim was a preliminary assessment of the possibility of extending the method to provide information on the degree of tumor infiltration at margins in IDH-mut gliomas.

### Method 1: Purdue University and Mayo Clinic.

**Fig. 2.**
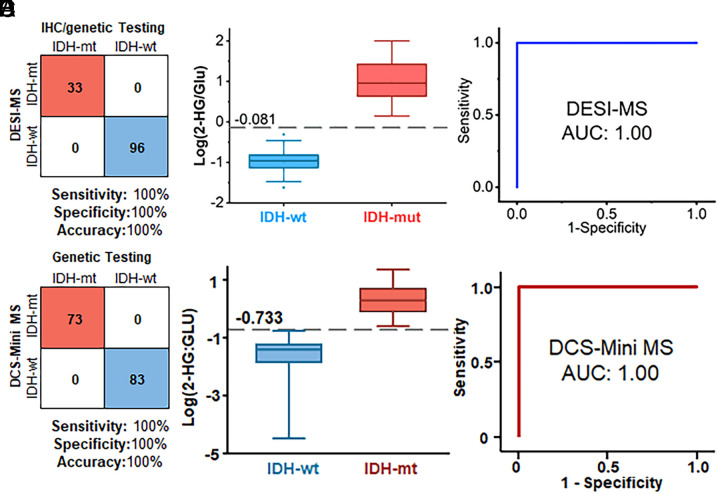
(*A*) Confusion matrix showing correlation between IHC and/or genetic testing and DESI-MS assessments of IDH genotype from 129 core biopsies from 31 patients. Intraoperative analysis of IDH mutation by 2HG/Glu achieved a sensitivity, specificity, and accuracy all of 100% by DESI-MS. (*B*) Box plots demonstrating separation of IDH-wt and IDH-mut tumors by DESI-MS measurements (***p < 0.0001). (*C*) ROC curves demonstrate perfect classification of IDH-wt and mut tumors by 2HG/Glu ratios irrespective of method. (*D*, *E*, and *F*) are parallel figures from intraoperative analysis of 156 core biopsies by miniature MS with sensitivity, specificity, and accuracy all at 100% and ***p < 0.0001.

The training dataset is composed of previously published data consisting of 148 biopsies from 43 patients (*SI Appendix*, Table S1) (39, 40). The validation dataset is composed of intraoperative DESI-MS/MS data from the analysis of 240 biopsies, including 129 core, 93 margin, and 18 tissues with unknown location from 34 patients (*SI Appendix*, Table S2). Due to the technical issues, the surgical team was not able to record MRI images of 18 tissue samples from three patients. Therefore, for the individual assessment of core and margin biopsies, those 18 samples were excluded and only included when all core and margins were combined. The validation patient cohort and DESI-MS results are summarized in *SI Appendix*, Tables S3–S5. A summary of the patients recruited, whether a patient was included or excluded from analysis, and the total number of biopsies analyzed is provided in *SI Appendix*, Tables S2 and S3. Patient demographics, diagnosis, other information are described in *SI Appendix*, Table S4. Validation of the predictions of IDH mutation status by DESI-MS was made by comparison with determinations of IDH mutation status made by standard immunohistochemistry (IHC) and/or genetic testing (Fig. 2*A*). Assessment of IDH mutation status of these 129 core biopsies indicated 100% sensitivity, 100% specificity, and 100% accuracy. Poorer performance was achieved when core and margin biopsies were combined (89% sensitivity, 100% specificity, and 96% accuracy). The results of DESI-MS assessment of IDH mutation status for all analyzed biopsies are included in *SI Appendix*, Table S5.

Despite the speed with which this measurement is made and the very small amount (5 to 10 mg) of tissue used, high-quality signals are obtained, as can be seen in the representative spectra of IDH-mut and IDH-wt biopsies shown in *SI Appendix*, Fig. S2. A statistically significant (Wilcoxon rank-sum test *P* < 0.0001) increase in the 2HG signal relative to the signal from Glu in tumor core biopsies is observed in patients with IDH-mut gliomas compared with patients with IDH-wt gliomas. This difference is apparent in the box plots (Fig. 2*B*) where the box represents the interquartile range with a median line and whiskers at ±1.5 SDs. The clear differentiation between IDH wild type and mutant is consistent with previous observations by the same research team (39, 40). The area under the curve (AUC) of the receiver operating characteristic (ROC) curve (Fig. 2*C*) is 1.00 for the classification of tumor core biopsies. A ROC optimized cutoff of the log 2HG/Glu ratio, −0.081, is represented as a dashed line in Fig. 2*B*. This cutoff is dependent on the instrumental parameters chosen.

A small number of margin biopsies (n = 9 of 93) were misclassified intraoperatively with respect to their confirmed IDH mutation status. Varying relative concentrations of 2HG were observed in different biopsies, even from locations in close proximity. Further, concentrations of 2HG decrease near surgical margins, rendering accurate detection more difficult. The presence of 2HG in tumor margin biopsies indicates that tumor margins extend beyond the surgical margin. This is to be expected due to the high degree of heterogeneity of glioma, an observation the team made in a prior clinical study (40). This is also an area of active research in the field of magnetic resonance spectroscopy (46). Adopting a similar method to that employed in the previous study, when averaging the 2HG/Glu ratios for all core biopsies from the same patient and using the averaged ratio to predict IDH mutation status, none of the patients were misclassified (40). This reiterates the heterogenous nature of gliomas and the importance of sampling multiple locations when attempting to molecularly characterize the tumor, an approach which is made possible by the rapid speed of DESI-MS analysis.

**Fig. 3.**
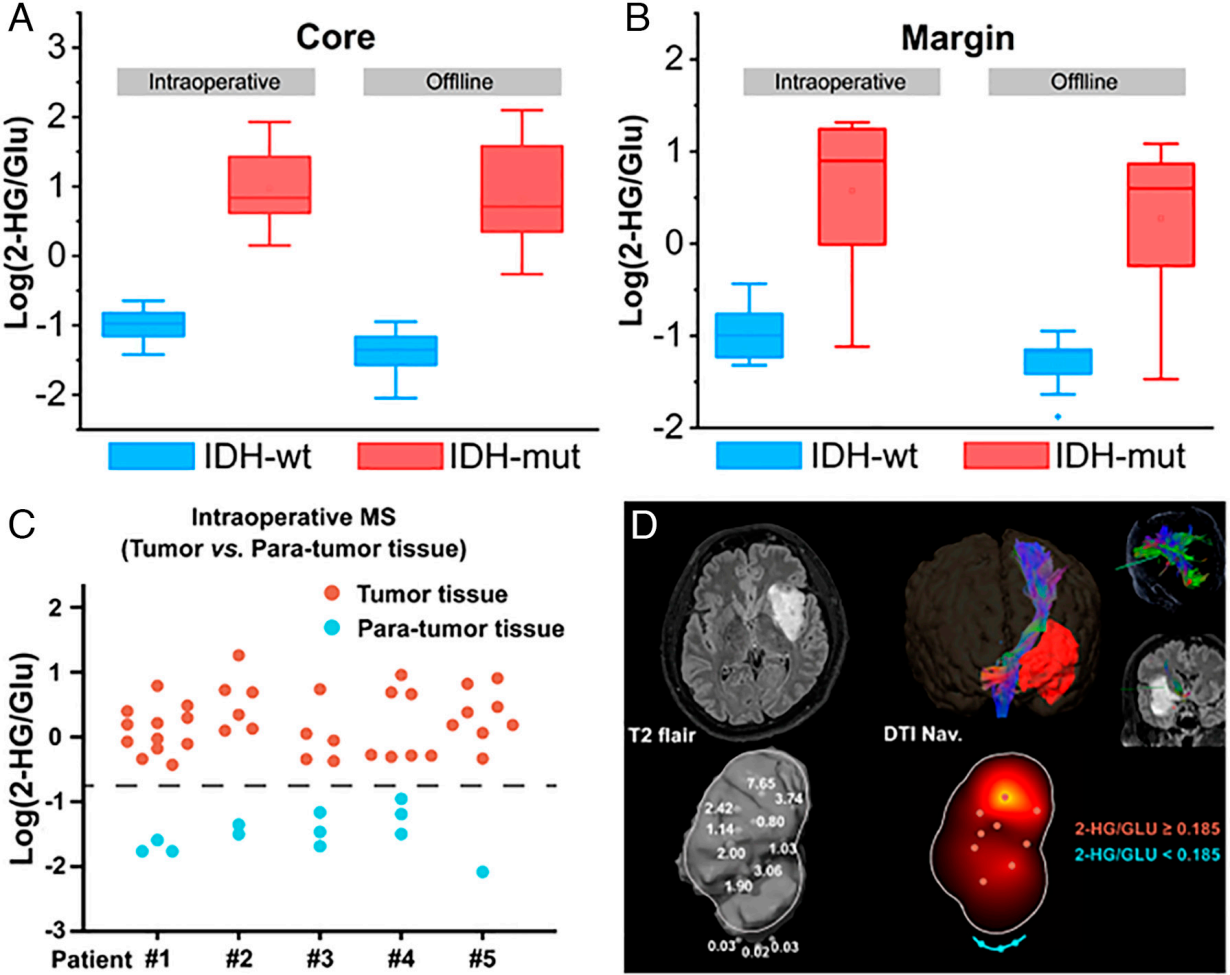
(*A* and *B*) Comparison of the intraoperative and postoperative assessment of IDH genotype from tumor core and surgical margin biopsies with DESI-MS. (*C*) Intraoperative metabolic analysis by 2HG/Glu with miniature MS could separate IDH mutated gliomas and paratumor (margin) tissue significantly (***P < 0.001). (*D*) Multiple sampling with navigation from a left insular glioma shows the intratumoral heterogeneity of 2HG. A metabolic borderline was delineated with consecutive sampling in real-time by 2HG/Glu analysis with miniature MS (blue line).

To assess the robustness of the DESI-MS/MS ratiometric method across multiple instruments, a small selection of biopsies (n = 82) was sent from Mayo Clinic to Purdue University where the measurement of 2HG/Glu was repeated on a laboratory Thermo LTQ using the same intraoperative methods. The comparison between the online and offline assessments of the IDH mutation status of the 82 biopsies is summarized in Fig. 3. Similar diagnostic performance was achieved for both the online and offline measurements of both core and margin biopsies, with poorer performance in margin biopsies hypothesized to be due to the heterogeneity in concentration of 2HG in the tumor volume potentially secondary to being in the supramarginal resection border (47–49). These data support the robustness of the method.

### Method 2: Tsinghua University and Huashan Hospital.

Samples of 137 biopsies from 109 patients including IDH-mut (n = 49), IDH-wt (n = 74), and noninfiltrated brain tissues (n = 14) were employed to train the miniature MS system for the diagnosis of IDH mutation. Relevant clinical information is shown in *SI Appendix*, Table S6. Signals for 2HG (*m/z* 147), Glu (*m/z* 146), and *m/z* 129 (itaconic acid or glutaric acid, ITA or GLA) were prominent in an S-plot distinguishing IDH-mut and IDH-wt biopsies by single-stage MS (*SI Appendix*, Fig. S3*A*). Differential expression of single-stage MS signals (MS1) for 2HG, Glu, and N-acetylaspartic acid (NAA) between gliomas with IDH-mut, IDH-wt, and normal brain tissue (*SI Appendix*, Fig. S3*B*) indicate 2HG accumulation in IDH mutated gliomas (*P* <0.0001). After normalization, the AUC value based on the relative abundance (MS1) of 2HG was 0.979 (*SI Appendix*, Fig. S3*C*). This value increased to 0.985 when using the ratio of 2HG to Glu. *SI Appendix*, Table S7 includes the cutoff value, sensitivity, specificity, Youden index, and AUC of different parameters for IDH mutation detection, comparing different MS methods of discrimination. The 2HG/Glu ratio produced the best discrimination and, therefore, was tested in the validation study.

The separation observed in the training dataset was tested in a prospective validation study using biopsies collected intraoperatively from 74 patients including 70 patients with gliomas and four without glioma (meningioma or neurilemmoma). The characteristics of the patients in the training and validation cohorts are compared in *SI Appendix*, Table S8, the cutoff value, sensitivity, specificity, Youden index, and AUC of different parameters for IDH mutation detection in the validation cohort are given in S *SI Appendix*, Table S9 and the tissue information, patients’ demographics, and clinical diagnosis are described in *SI Appendix*, Table S10. Note that the validation dataset (*SI Appendix*, Table S10) comprised 73 biopsies from IDH-mut gliomas (28 patients), 83 biopsies from IDH-wt gliomas (42 patients), and 16 adjacent paratumor biopsies to further verify the accuracy and reliability of the miniature MS system for intraoperative IDH mutation detection. The ion abundance ratio of 2HG/Glu was significantly higher, 0.243 to 22.749 in IDH-mut gliomas, compared to 0.00003 to 0.174 in IDH-wt tissues (*P* < 0.001), indicating that the cutoff of 0.185 (log −0.733 in Fig. 2*E*) could differentiate between IDH-wt and IDH-mut gliomas. The accuracy, sensitivity, and specificity for IDH mutation detection in core biopsies by 2HG/Glu (MS/MS) were all at 100% (Fig. 2*D*). The validation cohort also showed the consistent diagnostic value of 2HG/Glu for the assessment of IDH mutation status, which was better than 2HG alone, as the accuracy of 2HG/Glu (100%) was higher than that of 2HG (91.7%, 95% CI: 86.3 to 96.0%). This finding is consistent with that observed by the Purdue University and Mayo Clinic team.

## Discussion

The most important finding in this study is that intraoperative mass spectrometry measurements can reliably detect the IDH mutation status of gliomas. Both the Purdue University/Mayo Clinic and Tsinghua University/Huashan Hospital studies core biopsies gave 100% agreement with immunohistochemistry (IHC) and standard genomics assignments of IDH mut gliomas. In the Purdue University/Mayo Clinic study, 2HG concentrations indicative of IDH mutation scores above the ROC cutoff (i.e., 2HG concentrations higher than those recorded for noninfiltrated tissue) were detected in 80% of margin biopsies from IDH-mut tumors (35 of 44 biopsies). A similar finding was made in the Tsinghua University/Huashan Hospital component of the study. This is illustrated for multiple biopsies (9 to 15 biopsies per case) from five patients analyzed intraoperatively using multiple points guided by neuro-navigation (Fig. 3 *C* and *D*). In IDH-mutated tissues, the 2HG/Glu ratios are significantly higher in IDH-mut tissues compared to adjacent noninfiltrated (paratumor) tissues (*P* < 0.001). This indicates that the ratio 2HG/Glu clearly separates IDH-mut glioma areas from adjacent nondiseased brain and that the metabolic border of gliomas could be delineated in the case of IDH-mut tumors. This additional capability should allow elucidation of tumor tissue margins and hence influence maximal surgical resection, even though this application is limited to cases of IDH-mut gliomas.

In a recurrent case at Mayo Clinic, the clinical diagnosis indicated an oligodendroglioma with an undefined tumor grade. However, none of the biopsies showed any sign of IDH mutation when analyzed by DESI-MS. The offline MS measurements of the same biopsies agreed with the intraoperative DESI-MS results. This led to a request for further clinical investigation, which revealed that the tumor tissue was mostly radiation necrosis with minimal (if any) involvement by oligodendroglioma (*SI Appendix*, Fig. S4).

The potential value of intraoperative knowledge of IDH mutation status by MS was demonstrated in a confusing case of a patient with a bifrontal lesion (*SI Appendix*, Fig. S7). MS analysis of a preoperative needle biopsy of the lesion identified an IDH mutation with a log 2HG/Glu ratio of 0.27. At the time of surgery, frozen pathology relayed an initial intraoperative diagnosis of the lesion as lymphoma, a diagnosis which the attending neurosurgeon was skeptical of since IDH mutations are not typically present in lymphomas. Intraoperative MS analysis also identified an IDH-mutated glioma. Postoperative follow-up pathology provided a final diagnosis of an IDH mutated grade III oligodendroglioma. This case demonstrates the intraoperative advantage in this particular case of the MS method of detecting IDH mutations over pathological staining.

**Fig. 4.**
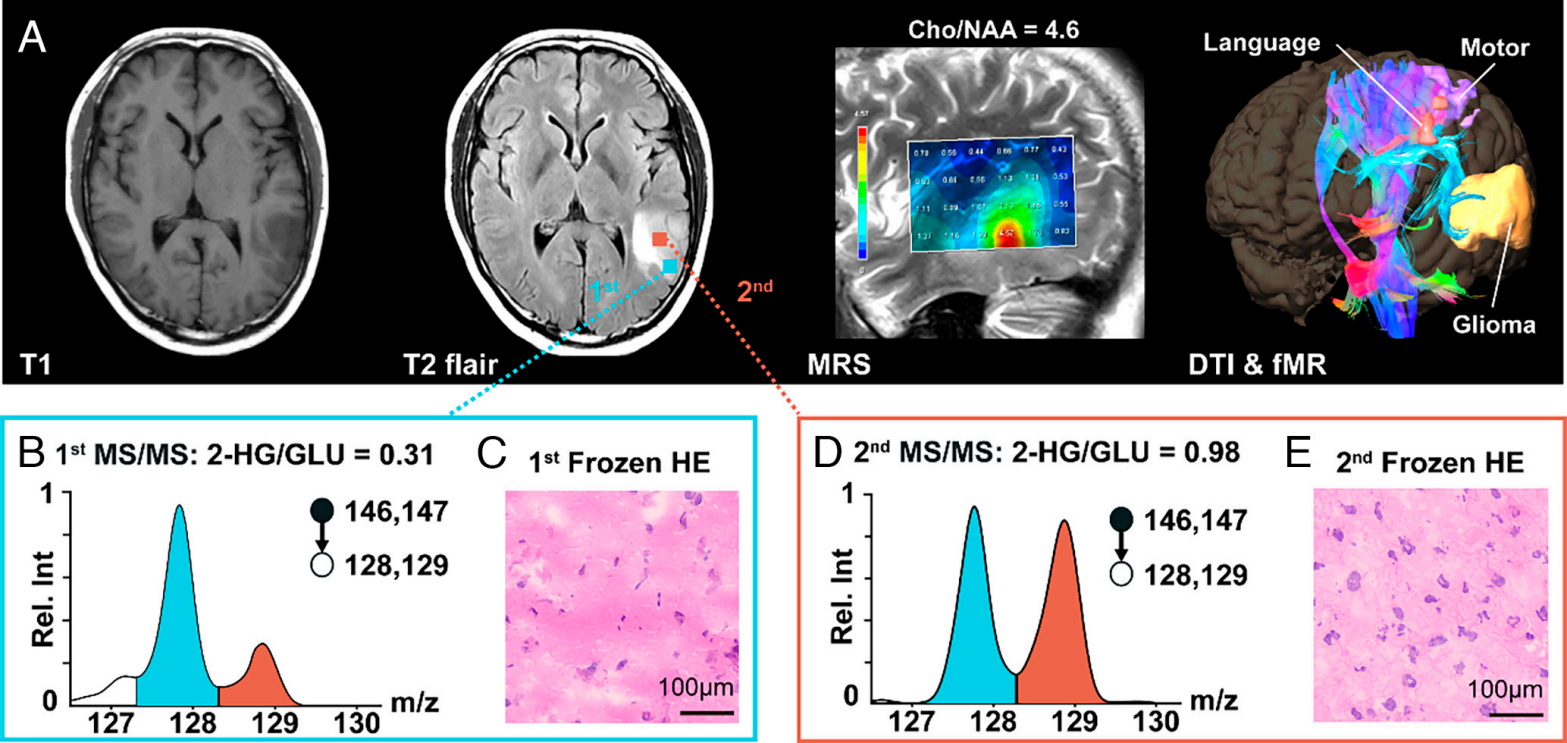
(*A*) Left posterior temporal lesion with a high Cho/NAA ratio on MRS was diagnosed as lower grade glioma preoperatively. (*B*) Miniature MS analysis revealed a high log 2HG/Glu ratio of 0.31, indicating the presence of an IDH mutation. (*C*) Frozen section reported gliosis with a low cell population. (*D*) Second MS/MS analysis revealed a higher log 2HG/Glu ratio of 0.98, indicating the presence of an IDH mutation. (*E*) Second pathological examination confirmed a lower-grade glioma.

The rapid, real-time miniature MS detection of IDH mutations is also valuable for distinguishing IDH-mut gliomas from gliosis, as was made apparent in other challenging cases (Fig. 4 and *SI Appendix*, Figs. S5–S7). For example, during the resection of a left temporal lobe glioma with a high Cho/NAA ratio using MRS (Fig. 4*A*), the first frozen section reported gliosis with a low cell population after examination of the slides obtained in 20 min, while the miniature MS system revealed a log 2HG/Glu ratio of 0.51 in 1.5 min, indicating the presence of an IDH mutation (Fig. 4 *B* and *C*). A second histological examination showed a typical lower-grade glioma, while the log ratio of 2HG/Glu (0.01) confirmed the presence of an IDH mutation (Fig. 4*D*). The postoperative staining and PCR sequencing confirmed the diagnosis of a grade II oligodendroglioma with IDH1 mutation and 1p/19q codeletion.

Further, with emerging evidence supporting the use of targeted therapy for IDH-mutant gliomas, the opportunity exists to intraoperatively diagnose IDH-mutations. This fact, coupled with its use to diagnose margin infiltration status and guide surgical resection, highlights the potential value of MS for therapeutic intervention in appropriate patients.

## Conclusion

Two independently developed and validated comprehensive MS systems for diagnosing IDH mutations intraoperatively are described in this study. Rapid detection was achieved within 2 min using methods compatible with the current surgical workflow. Ionization by DESI or DCS allows direct analysis of brain tissue while MS/MS minimizes interferences from isomers and isobars. IDH mutation status was determined with high accuracy and allowed correlation of 2HG accumulation with tumor infiltration. A strong correlation of high ratios of 2HG to Glu in IDH-mut gliomas (*P* <0.0001) was observed by both teams. Despite the use of different ionization methods and mass spectrometers, comparable performance in determining IDH mutations from core tumor biopsies was observed with sensitivities, specificities, and accuracies of 100%, regardless of IDH1 or IDH2 genotype. Further, both teams noticed the presence of high concentrations of 2HG at surgical margins. 2HG is a unique biomarker only present in IDH-mutant tumor, and this means that it could be used as a surrogate for tumor infiltration. Intraoperative information on IDH mutant tissue infiltration should allow surgeons to achieve better patient outcomes by directly influencing the extent of surgical resection.

## Methods

### Human Subjects.

Human subjects research was performed in accordance with individual Institutional Review Board (IRB) approvals for Mayo Clinic and Huashan hospital. All patients with suspected glioma undergoing craniotomy with tumor resection were enrolled after providing written informed consent. More information about human subjects can be found in *SI Appendix*, section S1.1. No clinical information from patient cohorts was shared between Mayo Clinic and Huashan Hospital.

### Method 1: Purdue University and Mayo Clinic.

Small tissue biopsies were provided by a neurosurgeon during craniotomy surgery. The location of the biopsies was decided at the surgeon’s discretion, with the intention of collecting biopsies from both the tumor core (for assessing IDH mutation status) and surgical margins (for assessing tumor infiltration). Individual biopsies (ca. 5 to 10 mg, each) were smeared on a glass slide, allowed to dry, and placed on the DESI stage; 5 kV was applied to nebulize a mixed solvent (DMF-ACN-EtOH, 25:37:38 by volume) for the extraction and ionization of analytes. The DESI-MS spray moved in a serpentine pattern in order to obtain representative DESI-MS data in the negative polarity (for details, see *SI Appendix*, section S1.3).

### Method 2: Tsinghua University and Huashan Hospital.

To perform integrated extraction and ionization, the tissue biopsy was touched with a thin strip of sampling paper which was then exposed to a solvent mixture (50 μL of ethanol/water, 9:1, v/v) in the sample cavity of a cartridge to extract analyte. The extract was then ionized by nESI using a spray voltage of 4.5 kV to generate ions (details in *SI Appendix*, section S1.4).

### Mass Analysis.

Assessment of IDH mutation status was achieved by simultaneous isolation of ions of *m/z* 146 and 147 and MS/MS analysis of their fragmentation products, *m/z* 147→129 (2HG) and *m/z* 146→128 (Glu). The contribution of the ^13^C isotope of Glu (6.1% of the major [Glu-H]^−^ signal) overlaps with the signal for 2HG (precursor *m/z* 147.1, product *m/z* 129.1) and was accounted for by subtracting its contribution to the detected signal at *m/z* 129 (*SI Appendix*, Fig. S1 and Eq. **S1**). A customized MATLAB algorithm and statistical test were used to process MS data (more information in *SI Appendix*, section S1.6). MS predictions of IDH mutation status were compared to IHC staining and/or PCR sequencing, the conventional gold standard methods to evaluate the methods’ performance. More information about IHC and PCR tests is provided in *SI Appendix*, section S1.2.

### Compliance with Ethical Standards.

For the work performed at Mayo Clinic in collaboration with Purdue University, biopsies for tissue analysis were obtained from human subjects undergoing tumor resection for suspected glioma at Mayo Clinic (Jacksonville, FL) after they had provided written informed consent to participate in the research study, following an IRB approved protocol (IRB No. 19-010725). No results were shared with the neurosurgeons during the surgical resection so as not to affect the standard of care. The work performed at Huashan Hospital in collaboration with Fudan University was approved by the ethics committee (IRB No. KY2019-587) and written informed consents were obtained by all patients. The diagnostic trial was registered in chictr.org.cn (ChiCTR2100044931).

## Supplementary Material

Appendix 01 (PDF)

Movie S1. **Video showing tissue collection and analysis by DESI-MS to illustrate the speed of mutation status determination**. A video showing the standard of care for the treatment of a patient suspected of glioma tumor undergoing craniotomy surgery. The sequential steps of the surgical resection are demonstrated. The freshly resected tissue samples from different locations of the tumor are transferred to the DESI-MS operator, and then, a small piece of each tissue is smeared on a glass slide by a 3D-printed smearer tool. The biopsy smear is placed on the DESI source for performing tandem MS measurements. The fragment ions of 2HG and Glu are simultaneously detected and measured in MS/MS mode. IDH-mut tumors show significantly higher 2HG signals compared to Glu.

## Data Availability

Some study data available [De-identified clinical data is provided in *SI Appendix*. Requests for additional de-identified clinical data will be entertained subject to legal and ethical considerations as guided by the lead clinicians (Dr. Alfredo Quinones-Hinojosa, Mayo Clinic and Dr. Ying Mao, Huashan Hospital). Mass spectrometry data beyond that provided in the manuscript and *SI Appendix* has been deposited in the Purdue University Research Repository (50). This data set comprises the full mass spectra and tandem mass spectra associated with analysis of all biopsies of representative patients].
