# Supplementary material for: Rapid detection of IDH mutations in gliomas by intraoperative mass spectrometry
Source: Proc Natl Acad Sci U S A. 2024 May 28;121(23):e2318843121. doi: 10.1073/pnas.2318843121 (PMC11161794; doi:10.1073/pnas.2318843121)
Supplement: Supplementary file 1 — Appendix 01 (PDF) [file pnas.2318843121.sapp.pdf]

Supporting Information

Rapid Detection of IDH Mutations in Gliomas by Intraoperative Mass Spectrometry

Wei Hua[#1,2,3,4,5], Wenpeng Zhang[#6] , Hannah Brown[#7], Junhan Wu[#6], Xinqi Fang[1,2,3,4,5], Mahdiyeh Shahi[7], Rong Chen[7], Haoyue Zhang[3], Bin Jiao[3], Nan Wang[8], Hao Xu[1,2,3,4,5], Minjie Fu[1,2,3,4,5], Xiaowen Wang[1,2,3,4,5], Jinsen Zhang[1,2,3,4,5], Xin Zhang[1,2,3,4,5], Qijun Wang[1,2,3,4,5], Wei Zhu[1,2,3,4,5], Dan Ye[9], Diogo Moniz Garcia[10], Kaisorn Chaichana[10], R. Graham Cooks*[7], Zheng Ouyang*[6], Ying Mao*[1,2,3,4,5], Alfredo Quinones-Hinojosa*[10]

[1] Department of Neurosurgery, Huashan Hospital, Fudan University, Shanghai, 200040, China.

[2] National Center for Neurological Disorders, Shanghai, 200040, China

[3] Shanghai Key Laboratory of Brain Function Restoration and Neural Regeneration, Shanghai, 200040, China

[4] Neurosurgical Institute of Fudan University, Shanghai, 200040, China

[5] Shanghai Clinical Medical Center of Neurosurgery, Shanghai, 200040, China

[6] State Key Laboratory of Precision Measurement Technology and Instruments, Department of Precision Instrument, Tsinghua University, Beijing, 100084, China

[7] Department of Chemistry, Purdue University, West Lafayette, IN 47907, US

[8] PURSPEC Technologies, Beijing, 100084, China

[9] The Molecular and Cell Biology Lab, Institute of Biomedical Sciences, Shanghai Medical College, Fudan University, Shanghai, 200232, China

[10] Department of Neurosurgery, Mayo Clinic, Jacksonville, FL 32224, US

#These authors contributed equally to this work.

To whom correspondence may be addressed. Email: maoying@fudan.edu.cn, ouyang@tsinghua.edu.cn, quinones-hinojosa.alfredo@mayo.edu, or cooks@purdue.edu

Table of Contents

S 1. Experimental 4

S 1.1. Human Subjects 4

S1.2. Diagnosis of IDH Genotype by IHC Staining and/or PCR Sequencing (both sites) 5

S 1.3. Method 1: DESI (Purdue University and Mayo Clinic) 5

S 1.4. Method 2: Direct capillary spray (DCS) (Tsinghua University and Huashan Hospital) . . . 7

S 1.5. Mass analysis and IDH score 8

S 1.6. Statistical Analysis (both sites) 8

Fig. S1. (A) Precursor ions for 2-HG, GLU, and its isotope in negative mode. **(B)** Single stage mass spectrum of GLU in negative mode (standard solution with GLU of 1 ppm). **(C)** Mass spectrum of a glioma tissue, and 2-HG peak after subtracting GLU isotope with red line.. 9

Fig. S2. Representative spectra of IDH-mut and IDH-wt biopsies obtained in MS/MS mode with DESI-MS. 10

Fig. S3. (A) S-plot diagram of metabolites to distinguish IDH-mut and IDH-wt gliomas was obtained by orthogonal projections to latent structures discriminant analysis (OPLS-DA) analysis. **(B)** Differential expression of 2HG, Glu, and NAA in IDH-mut and IDH-wt gliomas, and in non-infiltrated brain tissues. (***p<0.001) **(C)** ROC curves obtained using single stage MS relative intensity of metabolites for IDH mutation, as well as MS/MS data for 2HG/Glu and 2HG/NAA. AUC values are 0.839, 0.979 and 0.985, respectively. **(D)** Flow chart of the diagnosis of IDH mutation using the miniature MS and the ratio of 2HG/Glu in MS/MS of 0.185.. 10

Fig. S4. Case of a 62 year-old male with prior history of oligodendroglioma (IDH-mutant and 1p/19q co-deleted) showing status after two surgical resections, chemotherapy and radiation therapy (last exposure one year ago) with intervening growth of the residual lesion. **(A)** T1-weighted post-gadolinium magnetic resonance imaging (MRI) axial cut demonstrating amorphic enhancement in the right temporal and occipital region surrounding the atrium of the right lateral ventricle, again redemonstrated in **(B)** T2-weitghted MRI axial cut imaging demonstrating substantial vasogenic edema surrounding the lesion, most prominent anteriorly. (C and D) Mass spectra from online and offline analysis of a core tissue biopsy of the same patient showing minimal DH mutation. 11

Fig. S5. (A) T1, T2 flair, Contrast MR, DTI images of a right temporal glioma, **(B)** MS/MS spectrum shows a 2HG/Glu ratio of 6.5, indicating the presence of an IDH mutation, **(C)** HE and **(D)** *IDH1 R132H* immunohistochemical staining identify the sample as a grade 2, IDH1 R132H negative, diffused astrocytoma, and **(E)** PCR Sequencing identifies the sample as a *IDH2 R172K* mutant tumor.

Analysis of the frequency of different point mutations of *IDH1* **(F)** and *IDH2* **(G)** from *COSMIC* database. 12

Fig. S6. (A) T2 and MRS images of a right diffuse frontal and temporal lesion, which underwent biopsy with navigation, **(B)** Intraoperative MS/MS spectrum analysis showed high 2-HG/GLU, **(C)** Intraoperative frozen HE showed low grade glioma, **(D)** Postoperative H&E also showed low grade glioma and **(E)** immunohistochemical staining showed *IDH1 R132H* mutant. 13

Fig. S7. (A) T1, T2 flair, Contrast MR images of a bifrontal lesion, **(B)** Intraoperative MS/MS spectrum analysis showed high 2-HG/GLU, **(C)** Intraoperative frozen HE was highly suspicious of a lymphoma, **(D)** Postoperative H&E showed low grade glioma and **(E)** *IDH1 R132H* immunohistochemical staining confirmed a diagnosis of an IDH-mutated glioma. 14

Table S1. Clinical information and IDH scores for the training data set (Purdue University/Mayo Clinic study). 14

Table S2. Summary of patient numbers, biopsy locations and IDH mutation status in Purdue University/Mayo Clinic study 19

Table S3. Summary of patients recruited, if excluded from study or analysis, if biopsies were subsequently analyzed offline, total biopsies analyzed intraoperatively, and number of biopsies analyzed offline (Purdue University/Mayo Clinic study).. 20

Table S4. Patient demographics, diagnosis, recurrence, IDH genotype (Purdue University/Mayo Clinic Study). 21

Table S5. Biopsy location, MS/MS data, DESI-MS IDH mutation status prediction (Purdue University/Mayo Clinic) 23

Table S6. Clinical demographics, tumor diagnosis, Miniature MS results for patients in the training data set (Tsinghua University/Huashan Hospital study). 31

Table S7. Cutoff, sensitivity, specificity, Youden index and AUC of metabolites for the diagnosis of IDH mutation in the training cohort comparing different methods of MS discrimination (Tsinghua University/Huashan Hospital study).. 38

Table S8 Characteristics of patients in two cohorts (Tsinghua University/Huashan Hospital study) . 39

Table S9. Cutoff, sensitivity, specificity, and Youden Index of 2-HG/GLU ratio for the detection of IDH mutation in validation set (Tsinghua University/Huashan Hospital study). 39

Table S10. Clinical demographics, tumor diagnosis, and Miniature MS results for patients in the validation data set (Tsinghua University/Huashan Hospital study). 40

S.2. Conclusion . . . . . Error! Bookmark not defined.

Video showing tissue collection and analysis by DESI-MS to illustrate the speed of mutation status determination. 47

References 48

S 1. Experimental

S 1.1. Human Subjects

Human subjects research at Mayo Clinic was performed in accordance with an Institutional Review Board approved study (IRB #19-010725). The training data were acquired in earlier published studies (Table S1). Validation data were obtained for patients with suspected glioma undergoing craniotomy with tumor resection who were prospectively enrolled after providing written informed consent and Health Insurance Portability and Accountability Act authorization. Intraoperative DESI-MS results were not shared with the participating neurosurgeons so as not to affect the standard of care. In addition to intraoperative measurements of biopsies, additional biopsies were stored frozen (-80 °C) in a tissue bank for post-operative validation. The pathology and molecular diagnostic results were confirmed according to WHO CNS4 in the training cohort and WHO CNS5 in the validation cohort.

With respect to the study performed at Huashan Hospital, training and validation patient cohorts were recruited under supervision of an independent IRB at Huashan Hospital, Fudan University (KY2019-587) registered in chictr.org.cn (ChiCTR2100044931). The recruited group included adult patients with suspected gliomas who underwent neurosurgical resection or biopsy. The sample size was determined based on a statistical power analysis (1) so as to provide a sensitivity of 0.98 and specificity of 0.96. Marginal error of 95% and confidence interval (95%) was set as 0.05 for both sensitivity and specificity. The sample size needed to achieve this performance was at least 98 and a total of 109 patients were recruited for diagnostic testing. The training cohort included 104 glioma patients and 5 non-glioma patients from 2016 to 2020 and the prospective validation cohort included 74 patients (70 glioma and 4 non-glioma) from 2020 to 2022.

At Huashan Hospital, samples from the training cohort were examined as frozen biopsies from a tissue bank and samples from the validation cohort were examined intraoperatively. The pathology and molecular diagnostic results were confirmed according to WHO CNS4 in the training cohort and WHO CNS5 in the validation cohort.

S1.2. Diagnosis of IDH Genotype by IHC Staining and/or PCR Sequencing (both sites)

Predictions of IDH mutation status determined by MS were compared to IDH genotype determined by IHC staining and/or PCR sequencing, the conventional gold standard methods. IHC staining was carried out for IDH mutation (R132H). Glioma specimens were routinely immunostained with an anti-IDH1-R132H antibody (1:100, Clone H09, Dianova) according to the manufacturers' specifications. Genomic DNA was isolated from approximately 30 mg of unstained histological brain tumor tissue with DNAeasy Kit (DNeasy Blood & Tissue Kit, QIAGEN, CA). DNA fragments spanning exons 4 of *IDH1* and *IDH2* were amplified by polymerase chain reaction on the isolated genomic DNA and directly sequenced. The primers used for polymerase chain reaction (PCR) amplification were IDH1F: AGCTCTATATGCCATCACTGC, IDH1R: AACATGCAAAATCACATTATTGCC, IDH2F: AATTTTAGGACCCCCGTCTG, and IDH2R: CTGCAGAGACAAGAGGATGG. PCR conditions for *IDH1* and *IDH2* amplifications were identical. The conventional PCR was applied with denaturation at 94°C for 3 min, followed by 30 cycles of denaturation at 94°C for 30 s, annealing at 55°C for 30 s, and extension at 72°C for 30 s. At Huashan Hospital, sequencing was performed on a semi-automated sequencer (GeneAmp® PCR System 9700, Base Module, Applied Biosystems). At Mayo Clinic, IDH1 and 2 mutations were detected using the RotoGene Q, a real time PCR instrument that permits quantitative and qualitative detection of IDH mutations.

S 1.3. Method 1: DESI (Purdue University and Mayo Clinic)

Desorption electrospray ionization (DESI) is a label-free method in which a spray of charged (~5 kV) solvent microdroplets impacts a surface and extracts compounds which are then transported to the MS in the secondary droplets.(2) Desolvation and ionization of the analytes in the secondary droplets is followed by mass spectrometric analysis. An inherent benefit of DESI is that only charged droplets of solvent are sprayed, the secondary droplets containing analytes extracted from the tissue are collected directly by the mass analyzer, so there is no chance of capillary blockage as can occur in ESI-based methods.

Prior work (3–5) using samples obtained at Methodist Hospital, Indianapolis and the same custom modified mass spectrometer involved comparisons of different MS/MS methods (including MS^3) as well as a preliminary study using a miniature mass spectrometer. This led to the selection of MS/MS methodology employed in this study, viz. selection of the 2-HG and Glu precursor ions in the negative ion mode using a window of 3 mass/charge units and the recording of the ratio of the dehydration product ion abundances. All experiments were conducted using the same DESI-MS system using a modified linear ion trap mass spectrometer (Thermo LTQ) as previously described.(29, 30, 32) Prior to installation at Mayo Clinic, Jacksonville, the instrument (Thermo LTQ) was minimally modified to include additional sound-proofing materials lining the instrument cart to reduce the noise generated by the vacuum pumps. The instrument was subsequently moved from Purdue University to Mayo Clinic and installed in a hallway directly adjacent to the intraoperative MRI operating room. At the beginning of each case, the instrument was turned on and the performance was tested using a series of quality controls, specifically a tissue smear of IDH-mut and IDH-wt gliomas for the IDH mutation status and a smear of human brain tissue to assess the mass accuracy and signal intensity of the detection of lipids. Small stereotactic biopsies (ca. 5-10 mg, each) were provided by a neurosurgeon to the MS operators during tumor resection surgery. The number and location of the biopsies were decided at the surgeon's discretion, with the intention of providing eight to ten biopsies from both the tumor core (for assessing IDH mutation status) and surgical margins (for assessing tumor infiltration), whenever surgically safe. The locations of these biopsies were recorded using the "Snapshot" feature of the neuronavigation system (StealthStation, Medtronic). The tissue biopsies were smeared on a glass slide using a 3D printed smearer tool, allowed to dry, and rastered underneath the DESI-MS spray in a serpentine pattern in order to obtain representative DESI-MS data across the slide without regional bias. The MS/MS measurements of the targeted analytes for the assessment of IDH mutation status took 1.15 min. After the DESI-MS measurements were complete, tissue smears were transferred to a container filled with methanol for Hematoxylin and eosin (H & E) staining and pathology reading. The smears that were diagnosed as non-glioma (e.g., predominantly blood, normal, and necrotic) were excluded from the study. The locations

of two particular biopsies were initially determined to be core; however, in the final MRI reading, the assessment of the clinical expert assigned these two as tumor margin biopsies.

S 1.4. Method 2: Direct capillary spray (DCS) (Tsinghua University and Huashan Hospital)

In this study, the DCS method of micro-extraction(6) was used and the extract was examined by nESI, a common form of electrospray ionization that is well suited for intraoperative applications. The direct sampling cartridge integrates sample handling and electrospray ionization techniques, avoiding complicated sample transfer procedures during MS analysis. The cartridge consists of a sampling handle and a body case with an embedded fused silica capillary for electrospray. A strip of sampling paper was fixed onto the metal holder at the front of the sampling handle, which was used to collect tissue biopsies placed onto a glass slide. After sampling, the sampling handle was placed into the body case. The integrated cartridge and user-friendly operation software simplify the process without any required professional skills or background. High sensitivity is achieved for a wide range of metabolites by using electrospray-based direct sampling ion cartridge.

Samples in the training cohort were frozen tissue bank biopsies. Samples in the validation cohort were obtained intraoperatively in an advanced neurosurgical operating room with neuronavigation. For each case, a portion (~ 0.2 cm^3) of the tumor tissue was removed and placed on sterile gauze in the direct sampling cartridge and connected to the miniature MS. Simple, user-friendly intraoperative IDH detection software was developed to collect and analyze intraoperative raw data.

DCS ionization which has been demonstrated in the detection of several metabolites in tissue extracts.(7) The entire analysis process, including sampling, elution, and MS detection, was completed within 1.5 minutes (Figure 1(C)), showing a significant improvement over conventional LC-MS and GC-MS methods. The mass spectrometer used was a miniature linear ion trap mass spectrometer (Mini β, PURSPEC Technologies, Beijing, China). This system provided a mass resolution of 0.4 Da, enabling the accurate separation of target metabolites in the range *m/z* 50 - 300.

S 1.5. Mass analysis and IDH score

Targeted MS/MS was used by both research teams to determine the IDH -mutation status of gliomas. This relies on the simultaneous isolation and fragmentation of 2HG and Glu ions for calculation of an IDH mutation score. This was done by simultaneous isolation of ions of *m/z* 146 and 147 and MS/MS analysis of their fragmentation products, *m/z* 147→129 (2HG) and *m/z* 146→128 (Glu). The contribution of the ^{13}C isotope of Glu (6.1% of the major $[Glu\text{-}H]^-$ signal) overlaps with the signal for 2HG (precursor *m/z* 147.1, product *m/z* 129.1 and was accounted for by subtracting its contribution to the detected signal at m/z 129. The IDH mutation score (i.e., the MS/MS ion ratio corrected for the ^{13}C-Glu isotopic contribution) was calculated from one minute of MS/MS acquisition per sample, as shown in Equation 1, where I_{128} and I_{129} are absolute intensities of *m/z* 128 and 129 in the MS/MS spectrum.

$$IDH\ Mutation\ Score = log\frac{I_{129}-(I_{128}\times 6.1\%)}{I_{128}} \qquad \text{(Eq. S1)}$$

The use of the 2HG/Glu ratio to discriminate mutant from non-mutant tissue has the advantage that ions with similar mass (difference 1 Da) are being compared. In addition, IDH-mut gliomas usually show a transamination-dependent Glu biosynthesis deficit.(8) This amplifies the difference of the 2HG/Glu ratio between IDH-mut and IDH-wt samples.

S 1.6. Statistical Analysis (both sites)

De-identified clinical data, consisting of patient demographics and radiology, surgical, and pathology reports were obtained for each patient and allowed for correlation with the mass spectral data. All the data were independently verified by at least two trained staff for accuracy, completeness, and consistency. The raw MS data was preprocessed by normalization and filtering, peak-picking, and feature extracting using MATLAB (v2019b). A logarithmic IDH mutation score was calculated as the log ratio of the two product ion intensities after correcting for the isotopic contributions of the ^{13}C-Glu fragment ion at *m/z* 129. MetaboAnalyst5.0 (Method 1 and 2) was used to plot receiver operating characteristic (ROC) curves and estimate area under the curve (AUC) values. Delong tests was used to compare the different AUCs. Standard error of accuracy was estimated with the delta method for estimating 95% CI. All statistical analyses were completed using STATA (Method 1), the IBM SPSS

statistics version 26.0 (IBM Co., Armonk, NY, USA) (Method 2), or the MedCalc statistical software version 11.7 (MedCalc Software Ltd, Ostend, Belgium) (Method 2); $p < 0.05$ was set as significant for the Mann-Whitney U test and Chi-square test.

Specific to Method 2, all 8,686 brain tumor files were downloaded from the COSMIC database (Catalogue of Somatic Mutations in Cancer, cancer.sanger.ac.uk/cosmic). Extraction of detailed mutation data followed by IDH1/2 mutation frequency analysis was performed using R (version 4.0.2). The metabolites in normal and IDH-mut and IDH-wt after structural identification by MS/MS were drawn using OriginPro.

A

Metabolites	Precursor Ion $(M\text{-}H)^-$
GLU	146
GLU (Isotope)	147 (6.1%)
2-HG	147

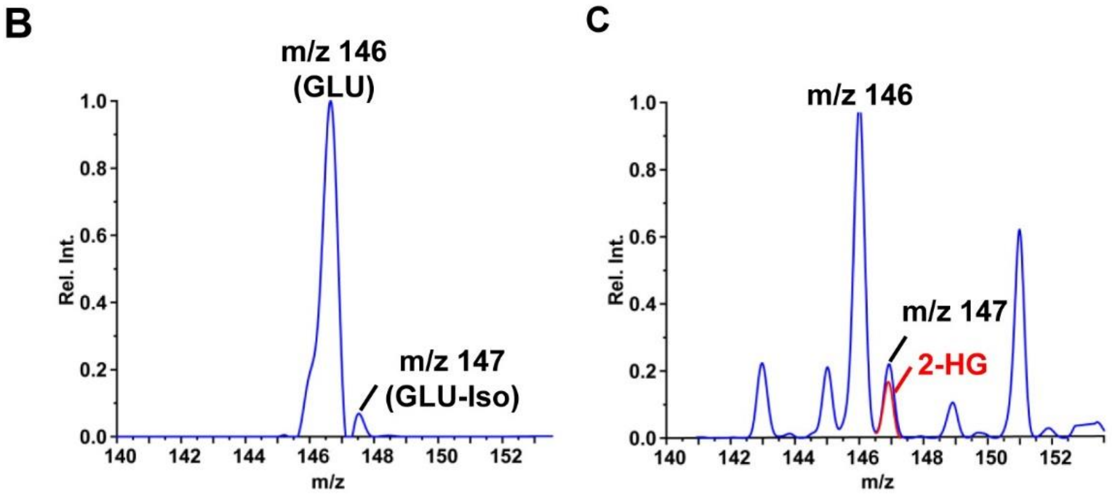

Fig. S1. (A) Precursor ions for 2-HG, GLU, and its isotope in negative mode. **(B)** Single stage mass spectrum of GLU in negative mode (standard solution with GLU of 1 ppm). **(C)** Mass spectrum of a glioma tissue showing 2-HG peak in red after subtracting GLU ^{13}C isotope contribution.

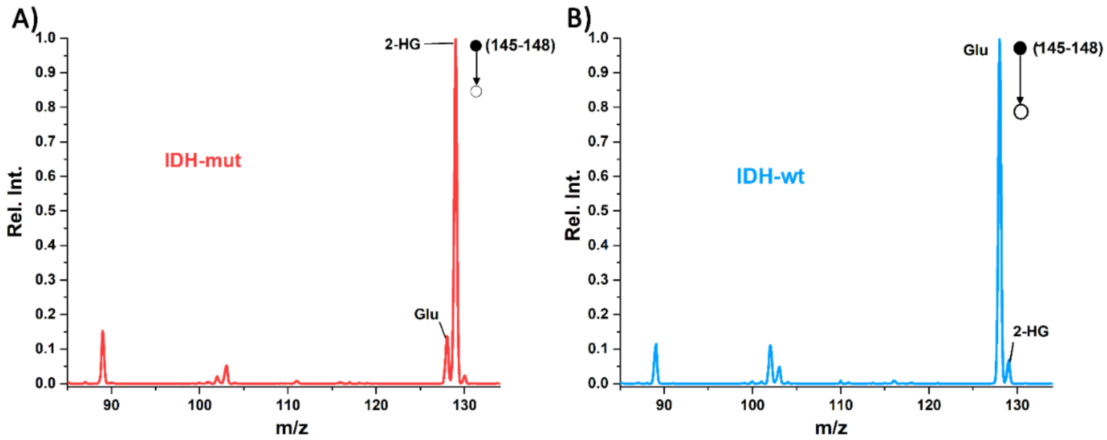

Fig. S2. Representative spectra of IDH-mut and IDH-wt biopsies obtained in MS/MS mode with DESI-MS.

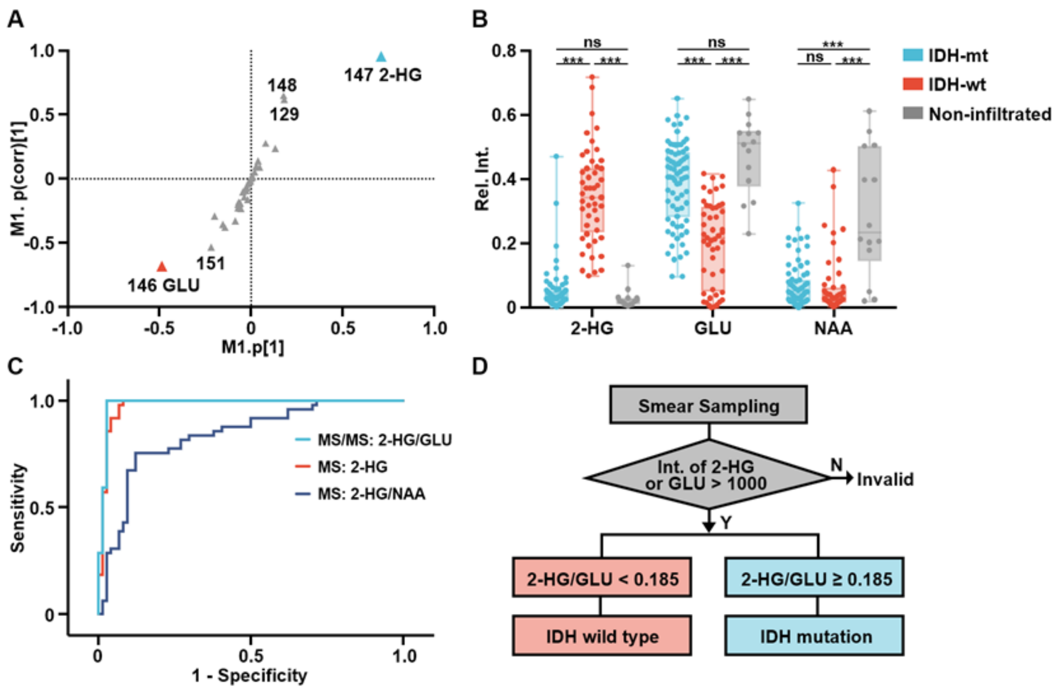

Fig. S3. (A) S-plot diagram of metabolites to distinguish IDH-mut and IDH-wt gliomas was obtained by orthogonal projections to latent structures discriminant analysis (OPLS-DA) analysis. **(B)** Differential expression of 2HG, Glu, and NAA in IDH-mut and IDH-wt gliomas, and in non-infiltrated brain tissues. (***$p<0.001$) **(C)** ROC curves obtained using single stage MS relative intensity of metabolites for IDH mutation, as well as MS/MS data for 2HG/Glu and 2HG/NAA. AUC values are 0.839, 0.979 and 0.985, respectively. **(D)** Flow chart of the diagnosis of IDH mutation using the miniature MS and the ratio of 2HG/Glu in MS/MS of 0.185.

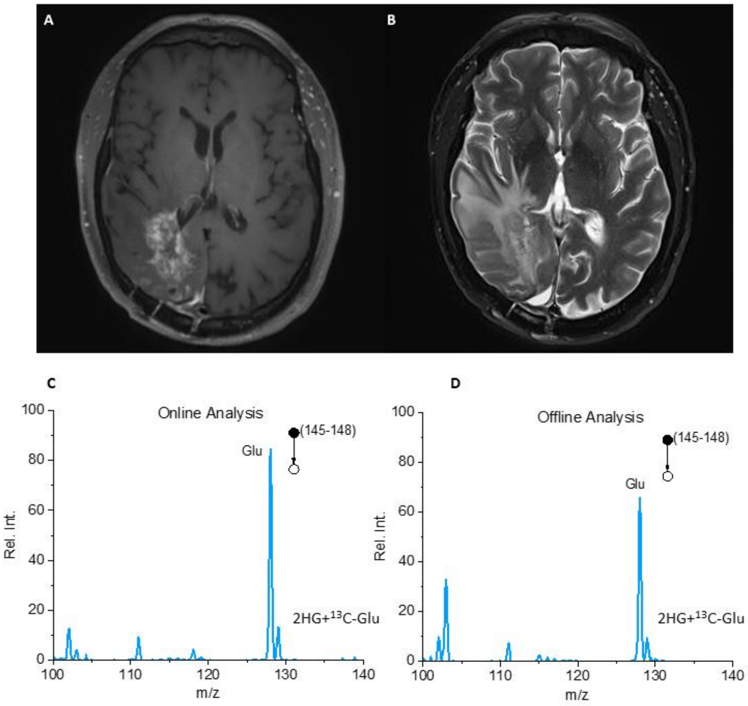

Fig. S4. Case of a 62 year-old male with prior history of oligodendroglioma (IDH-mutant and 1p/19q co-deleted) showing status after two surgical resections, chemotherapy and radiation therapy (last exposure one year ago) with intervening growth of the residual lesion. **(A)** T1-weighted post-gadolinium magnetic resonance imaging (MRI) axial cut demonstrating amorphic enhancement in the right temporal and occipital region surrounding the atrium of the right lateral ventricle, again redemonstrated in **(B)** T2-weitghted MRI axial cut imaging demonstrating substantial vasogenic edema surrounding the lesion, most prominent anteriorly. **(C and D)** Mass spectra from online and offline analysis of a core tissue biopsy of the same patient showing minimal DH mutation.

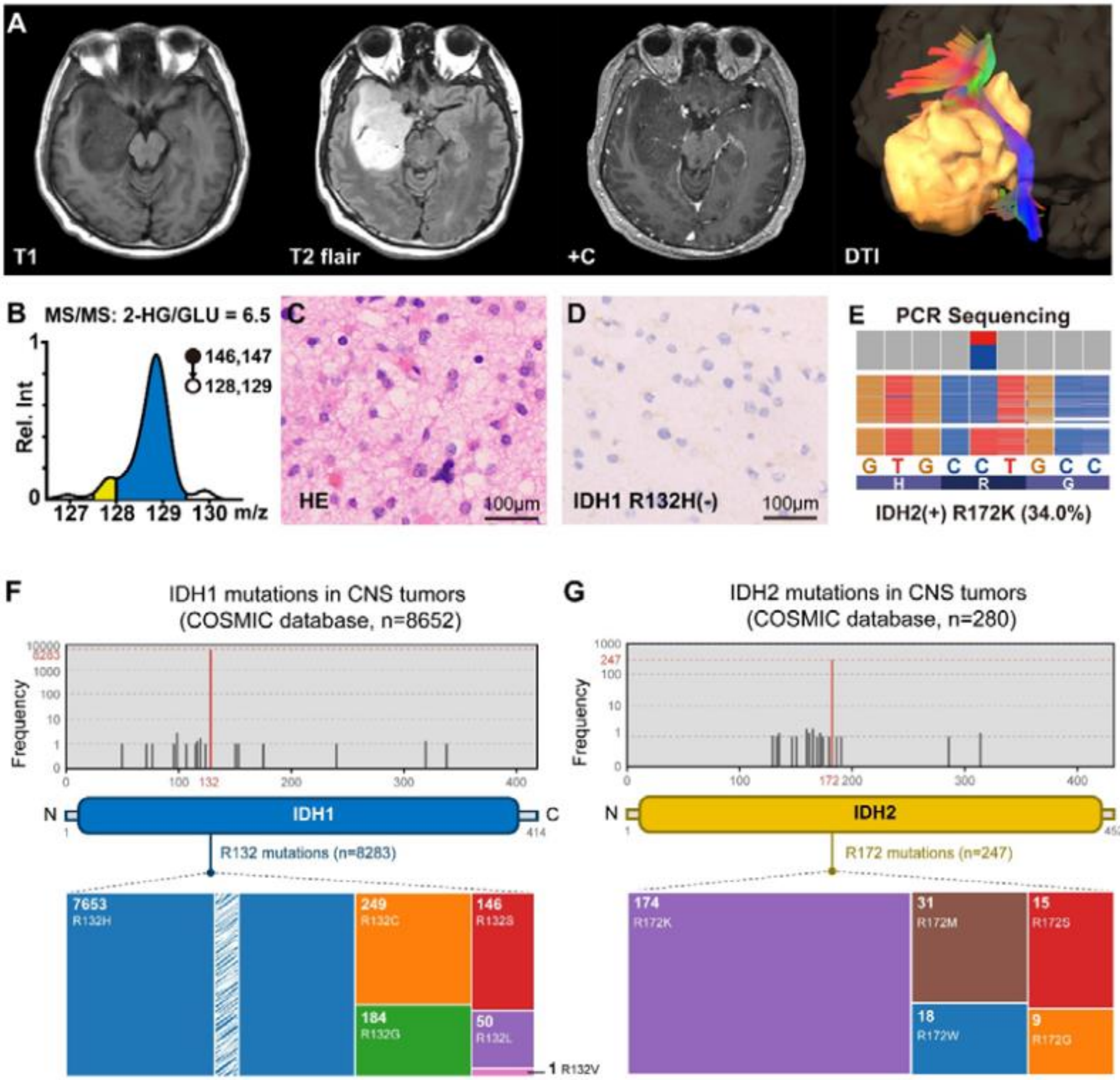

Fig. S5. (A) T1, T2 flair, Contrast MR, DTI images of a right temporal glioma, **(B)** MS/MS spectrum shows a 2HG/Glu ratio of 6.5, indicating the presence of an IDH mutation, **(C)** HE and **(D)** *IDH1 R132H* immunohistochemical staining identify the sample as a grade 2, IDH1 R132H negative, diffused astrocytoma, and **(E)** PCR Sequencing identifies the sample as a *IDH2 R172K* mutant tumor. Analysis of the frequency of different point mutations of *IDH1* **(F)** and *IDH2* **(G)** from *COSMIC* database.

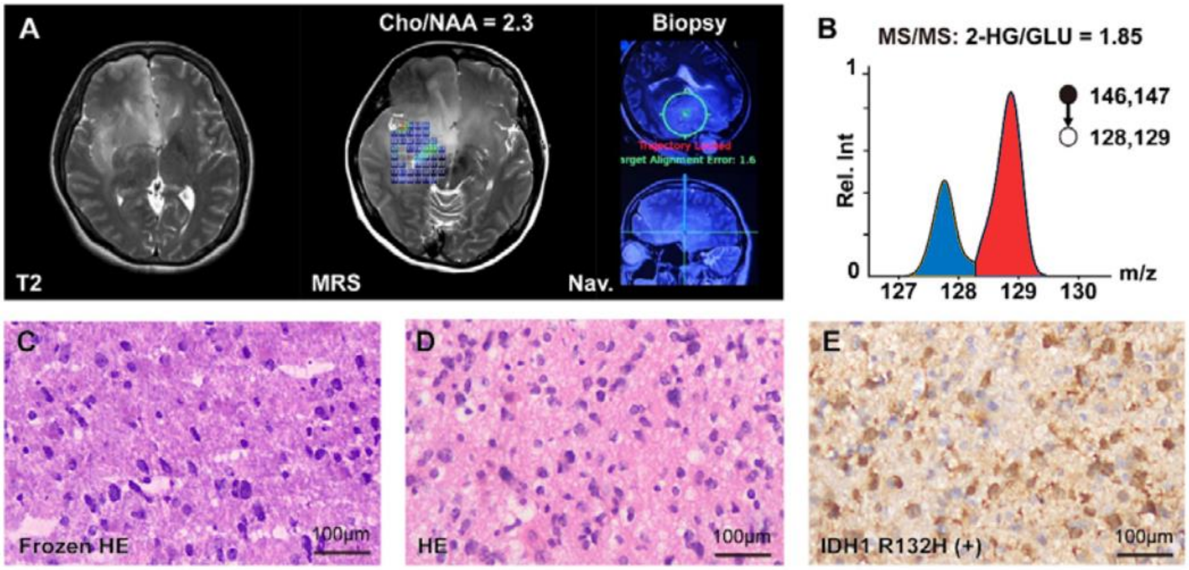

Fig. S6. (A) T2 and MRS images of a right diffuse frontal and temporal lesion, which underwent biopsy with navigation, **(B)** Intraoperative MS/MS spectrum analysis showed high 2-HG/GLU, **(C)** Intraoperative frozen HE showed low grade glioma, **(D)** Postoperative H&E also showed low grade glioma and **(E)** immunohistochemical staining showed *IDH1 R132H* mutant.

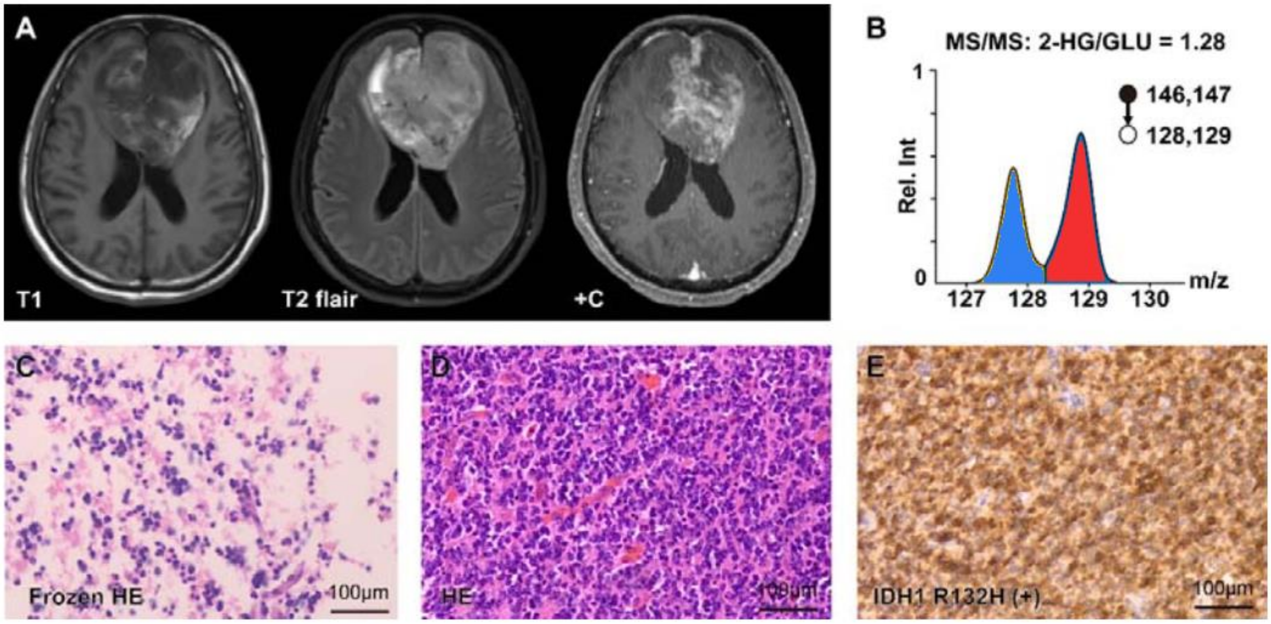

Fig. S7. (A) T1, T2 flair, Contrast MR images of a bifrontal lesion, **(B)** Intraoperative MS/MS spectrum analysis showed high 2-HG/GLU, **(C)** Intraoperative frozen HE was highly suspicious of a lymphoma, **(D)** Postoperative H&E showed low grade glioma and **(E)** *IDH1 R132H* immunohistochemical staining confirmed a diagnosis of an IDH-mutated glioma.

Table S1. Clinical information and IDH scores for the training data set (Purdue University/Mayo Clinic study)

Biopsy Number (overall)	General Subject ID	Biopsy Number (subject)	Location	Type of Glioma	WHO Grade	IDH Status	Instrument	MS3 Sum	IDH Ratio	Ref.
1	1	1	M	Glioblastoma	IV	WT	LTQ	2.70	-	(5)
2	1	2	C	Glioblastoma	IV	WT	LTQ	6.00	-	(5)
3	2	1	M	Glioblastoma	IV	WT	LTQ	0.00	-	(5)
4	2	5	M	Glioblastoma	IV	WT	LTQ	0.00	-	(5)
5	2	7	M	Glioblastoma	IV	WT	LTQ	8.20	-	(5)
6	3	1	C	Anaplastic astrocytoma	III	M	LTQ	26.00	-	(5)
7	3	2	M	Anaplastic astrocytoma	III	M	LTQ	61.40	-	(5)
8	3	3	M	Anaplastic astrocytoma	III	M	LTQ	70.50	-	(5)
9	3	4	M	Anaplastic astrocytoma	III	M	LTQ	82.50	-	(5)
10	3	5	C	Anaplastic astrocytoma	III	M	LTQ	62.30	-	(5)
11	4	1	C	Gliosarcoma	IV	WT	LTQ	16.90	-	(5)
12	4	2	M	Gliosarcoma	IV	WT	LTQ	0.00	-	(5)
13	4	3	M	Gliosarcoma	IV	WT	LTQ	30.60	-	(5)
14	4	4	C	Gliosarcoma	IV	WT	LTQ	8.20	-	(5)
15	5	1	C	Diffuse astrocytoma	II	WT	LTQ	0.00	-	(5)
16	5	2	M	Diffuse astrocytoma	II	WT	LTQ	0.00	-	(5)
17	5	3	M	Diffuse astrocytoma	II	WT	LTQ	10.50	-	(5)
18	5	4	C	Diffuse astrocytoma	II	WT	LTQ	0.00	-	(5)
19	5	5	C	Diffuse astrocytoma	II	WT	LTQ	21.90	-	(5)
20	5	6	M	Diffuse astrocytoma	II	WT	LTQ	4.70	-	(5)
21	6	1	C	Diffuse astrocytoma	II	WT	LTQ	47.00	-	(5)
22	6	2	M	Diffuse astrocytoma	II	WT	LTQ	12.60	-	(5)
23	6	3	M	Diffuse astrocytoma	II	WT	LTQ	0.00	-	(5)
24	6	4	M	Diffuse astrocytoma	II	WT	LTQ	56.20	-	(5)
25	6	5	M	Diffuse astrocytoma	II	WT	LTQ	26.50	-	(5)

26	7	1	M	Glioblastoma	IV	WT	LTQ	2.30	-	(5)
27		2	C				LTQ	0.00	-	(5)
28		3	M				LTQ	0.00	-	(5)
29		4	C				LTQ	0.00	-	(5)
30		5	C				LTQ	0.00	-	(5)
31	8	1	M	Glioblastoma	IV	WT	LTQ	0.00	-	(5)
32		2	C				LTQ	0.00	-	(5)
33		5	C				LTQ	0.00	-	(5)
34	9	2	M	Diffuse astrocytoma	II	M	LTQ	5.50	-	(5)
35		3	M				LTQ	59.70	-	(5)
36		4	C				LTQ	87.60	-	(5)
37		5	C				LTQ	43.80	-	(5)
38		6	C				LTQ	71.40	-	(5)
39	10	1	M	Glioblastoma	IV	WT	LTQ	3.65	-	(5)
40		2	C				LTQ	4.65	-	(5)
41		3	C				LTQ	5.25	-	(5)
42	11	1	C	Glioblastoma	IV	WT	LTQ	0.00	-	(5)
43		2	M				LTQ	22.40	-	(5)
44		3	M				LTQ	0.00	-	(5)
45		4	M				LTQ	0.00	-	(5)
46		5	M				LTQ	0.00	-	(5)
47		6	M				LTQ	12.90	-	(5)
48	12	1	C	Glioblastoma	IV	WT	LTQ	0.00	-	(5)
49		2	C				LTQ	0.00	-	(5)
50		3	C				LTQ	0.00	-	(5)
51		4	C				LTQ	0.00	-	(5)
52	13	1	C	Anaplastic astrocytoma	III	M	LTQ	33.50	-	(5)
53		3	C				LTQ	101.70	-	(5)
54		5	M				LTQ	15.10	-	(5)
55		6	C				LTQ	73.50	-	(5)
56	14	1	M	Glioblastoma	IV	WT	LTQ	38.30	-	(5)
57		2	M				LTQ	16.30	-	(5)
58		3	C				LTQ	19.70	-	(5)
59	15	1	M	Glioblastoma	IV	WT	LTQ	16.45	-	(5)

60		2	C				LTQ	29.70	-	(5)
61		3	M				LTQ	41.45	-	(5)
62	16	1	M	Diffuse glioma	-	M	LTQ	90.20	-	(5)
63		3	M				LTQ	26.10	-	(5)
64		4	M				LTQ	10.10	-	(5)
65	17	1	C	Astrocytoma	IV	M	LTQ	71.00	-	(5)
66		2	C				LTQ	180.30	-	(5)
67		3	C				LTQ	88.90	-	(5)
68	18	1	C	Diffuse astrocytoma	II	M	LTQ	86.40	-	(5)
69		2	C				LTQ	76.40	-	(5)
70		3	M				LTQ	79.40	-	(5)
71		4	M				LTQ	83.40	-	(5)
72		5	M				LTQ	79.30	-	(5)
73		6	C				LTQ	85.70	-	(5)
74	19	1	C	Diffuse glioma	-	WT	LTQ	58.75	-	(5)
75		2	C				LTQ	4.90	-	(5)
76		3	C				LTQ	40.57	-	(5)
77		4	M				LTQ	40.60	-	(5)
78		5	M				LTQ	29.00	-	(5)
79		6	M				LTQ	56.10	-	(5)
80	20	1	C	Anaplastic astrocytoma	III	M	LTQ	47.20	-	(5)
81		2	C				LTQ	107.30	-	(5)
82		3	C				LTQ	87.25	-	(5)
83		4	M				LTQ	70.45	-	(5)
84	21	1	C	Glioblastoma	IV	WT	LTQ	8.40	-	(5)
85		2	C				LTQ	30.55	-	(5)
86		3	C				LTQ	16.23	-	(5)
87	22	1	C	Diffuse astrocytoma	II	M	LTQ	106.70	-	(5)
88		2	M				LTQ	62.80	-	(5)
89		3	C				LTQ	154.50	-	(5)
90		4	C				LTQ	116.90	-	(5)
91		5	C				LTQ	76.90	-	(5)
92		6	C				LTQ	89.80	-	(5)
93	23	1	C	Astrocytoma	IV	M	LTQ	393.90	-	(5)

94		2	M				LTQ	466.20	-	(5)
95		3	M				LTQ	631.80	-	(5)
96		4	C				LTQ	657.60	-	(5)
97		5	M				LTQ	683.80	-	(5)
98		6	C				LTQ	524.10	-	(5)
99	24	1	M	Glioblastoma	IV	WT	LTQ	0.00	-	(5)
100		2	M				LTQ	28.60	-	(5)
101		3	C				LTQ	53.20	-	(5)
102	25	1	C	Diffuse astrocytoma	II	M	LTQ	113.10	-	(5)
103		2	M				LTQ	144.45	-	(5)
104		3	C				LTQ	140.35	-	(5)
105		4	C				LTQ	135.90	-	(5)
106		5	C				LTQ	195.30	-	(5)
107		6	M				LTQ	50.15	-	(5)
108	26	1	M	Astrocytoma	IV	M	LTQ	97.50	-	(5)
109		2	C				LTQ	220.70	-	(5)
110		3	C				LTQ	140.53	-	(5)
111	27	1	M	Glioblastoma	IV	WT	LTQ	9.20	-	(5)
112		2	C				LTQ	0.00	-	(5)
113		3	C				LTQ	0.00	-	(5)
114	28	1	C	Glioblastoma	IV	WT	LTQ	7.90	-	(5)
115		2	C				LTQ	45.87	-	(5)
116	29	1	C	Oligodendroglioma	II	M	LTQ	403.10	-	(5)
117		2	C				LTQ	517.73	-	(5)
118	30	1	C	Oligodendroglioma	II	M	LTQ	611.55	-	(5)
119		2	M				LTQ	453.50	-	(5)
120		3	M				LTQ	125.10	-	(5)
121		4	C				LTQ	247.90	-	(5)
122		5	M				LTQ	72.50	-	(5)
123		6	C				LTQ	436.40	-	(5)
124	31	1	C	Glioblastoma	IV	WT	Mini | TSQ	-	0.06 | <0.01	(9)
125	32	1	C	Glioblastoma	IV	WT	Mini | TSQ	-	0.02 | 0.02	(9)

126	33	1	C	Astrocytoma	II	M	Mini | TSQ	-	3.89 | 10.56	(9)
127	34	1	C	Pleomorphic xantoastrocytoma	II	WT	Mini | TSQ	-	0.33 | 0.03	(9)
128		2	C				Mini | TSQ	-	0.18 | 0.02	(9)
129		3	C				Mini | TSQ	-	0.35 | 0.04	(9)
130	35	1	C	Glioblastoma	IV	WT	Mini | TSQ	-	0.20 | 0.06	(9)
131		2	C				Mini | TSQ	-	0.24 | 0.06	(9)
132		3	C				Mini | TSQ	-	0.33 | 0.12	(9)
133	36	1	C	Glioblastoma	IV	WT	Mini | TSQ	-	0.35 | 0.04	(9)
134		2	C				Mini | TSQ	-	0.33 | 0.04	(9)
135	37	1	C	Glioblastoma	IV	WT	Mini | TSQ	-	0.28 | 0.07	(9)
136		2	C				Mini | TSQ	-	0.20 | 0.09	(9)
137	38	1	C	Anaplastic astrocytoma	III	M	Mini | TSQ	-	2.83 | 5.01	(9)
138	39	1	C	Glioblastoma	IV	WT	Mini | TSQ	-	0.11 | 0.05	(9)
139	40	1	C	Astrocytoma	IV	M	Mini | TSQ	-	3.20 |35.51	(9)
140		2	C				Mini | TSQ	-	1.73 |99.33	(9)
141		3	C				Mini | TSQ	-	1.73 |14.34	(9)
142	41	1	C	Glioblastoma	IV	WT	Mini | TSQ	-	0.12 | 0.06	(9)
143	42	1	C	Anaplastic astrocytoma	III	M	Mini | TSQ	-	2.07 |6.73	(9)

144		2	C				Mini | TSQ	-	2.30 |16.93	(9)
145	43	1	C	Glioblastoma	IV	WT	Mini | TSQ	-	0.25 |0.04	(9)
146		2	C				Mini | TSQ	-	0.35 |0.04	(9)
147		3	C				Mini | TSQ	-	0.50 |0.07	(9)
148		4	C				Mini | TSQ	-	0.26| 0.03	(9)

C= Core; M= Margin   # Note that WHO CNS5 nomenclature is used in this Table.

Table S2. Summary of patient numbers, biopsy locations and IDH mutation status in Purdue University/Mayo Clinic study

IDH Status	# Patients	# Core Biopsies	# Margin Biopsies	# Unknown Location	
**IDH-mut**	**11**	**33**	**44**	**6**	**Total IDH-mut: 83**
**IDH-wt**	**23**	**96**	**49**	**12**	**Total IDH-wt: 157**
	**Total Patients: 34**	**Total: 129**	**Total: 93**	**Total: 18**	
		**Total: 240**			

Table S3. Summary of patients recruited, if excluded from study or analysis, if biopsies were subsequently analyzed offline, total biopsies analyzed intraoperatively, and number of biopsies analyzed offline (Purdue University/Mayo Clinic study).

Patient #	Screenfail	Analyzed offline	Total Biopsies Analyzed	Biopsies Analyzed Offline
1	No	No	8	0
2	No	No	8	0
3	No	No	8	0
4	No	No	8	0
5	No	No	8	0
6	No	No	8	0
7	No	No	8	0
8	No	No	8	0
9	No	No	8	0
10	Yes	-	-	-
11	No	No	8	0
12	No	No	8	0
13	Yes	-	-	-
14	No	Yes	8	5
15	No	Yes	8	5
16	No	Yes	8	5
17	No	Yes	8	5
18	No	No	8	0
19	No	Yes	8	4
20	No	Yes	8	5
21	No	Yes	8	3
22	No	Yes	8	2
23	No	Yes	8	0
24	Yes	-	-	-
25	Yes	-	-	-
26	Yes	-	-	-
27	Yes	-	-	-
28	No	Yes	8	5
29	No	Yes	8	3
30	No	No	8	0
31	Yes	-	-	-

32	Yes	-	-	-
33	Yes	-	-	-
34	No	No	8	0
35	Yes	-	-	-
36	No	Yes	8	5
37	No	No	4	0
38	No	Yes	8	5
39	No	Yes	8	6
40	No	Yes	8	5
41	No	Yes	8	6
42	No	Yes	8	5
43	No	Yes	8	7
44	No	Yes	8	3

-= Information is irrelevant, as the patient was a screen fail

Table S4. Patient demographics, diagnosis, recurrence, IDH genotype (Purdue University/Mayo Clinic Study)

Patient #	Age	Gender	Race	Ethnicity	Glioma Classification	WHO Grade	Primary/ Recurrent	IDH Genotype
1	64	M	W	N.H.	Glioblastoma	IV	R	WT
2	46	F	W	H	Glioblastoma	IV	P	WT
3	59	M	W	N.H.	Glioblastoma	IV	P	WT
4	66	M	-	-	Glioblastoma	IV	P	WT
5	44	M	W	N.H.	Oligodendroglioma	II	-	Mut
6	46	M	W	N.H.	Glioblastoma	IV	R	WT
7	70	M	W	N.H.	Glioblastoma	IV	P	WT
8	47	M	W	N.H.	Astrocytoma	IV	R	Mut
9	-	M	-	-	Glioblastoma	IV	-	WT
10	53	F	W	N.H.	Glioblastoma	IV	R	WT
11	51	M	W	N.H.	Glioblastoma	IV	P	WT
12	72	M	W	N.H.	Astrocytoma	II	P	WT
13	20	F	W	N.H.	Astrocytoma	II	P	Mut
14	26	M	B	N.H.	Anaplastic astrocytoma	III	P	Mut
15	49	F	W	N.H.	Astrocytoma	II	P	Mut
16	71	M	W	N.H.	Glioblastoma	IV	R	WT
17	51	M	W	N.H.	Glioblastoma	IV	P	WT
18	37	F	A.I.	N.H.	Oligodendroglioma	II	P	Mut

19	60	F	W	N.H.	Glioblastoma	IV	P	WT
20	63	F	W	N.H.	Glioblastoma	IV	P	WT
21	71	M	W	N.H.	Anaplastic oligodendroglioma	III	P	Mut
22	81	M	W	N.H.	Glioblastoma	IV	P	WT
23	58	M	W	N.H.	Glioblastoma	IV	P	WT
24	39	M	W	N.H.	Astrocytoma	II	P	Mut
25	42	M	W	N.H.	Astrocytoma	II	P	Mut
26	82	F	W	N.H.	Glioblastoma	IV	P	WT
27	70	M	W	N.H.	Glioblastoma	IV	P	WT
28	62	M	W	N.H.	Oligodendroglioma	NA	R	*
29	27	M	W	H	Astrocytoma	III	P	Mut
30	70	F	W	N.H.	Glioblastoma	IV	P	WT
31	69	M	W	N.H.	Glioblastoma	IV	R	WT
32	58	M	W	N.H.	Glioblastoma	IV	P	WT
33	51	F	W	H	Astrocytoma	III	P	Mut
34	73	M	W	H	Glioblastoma	IV	P	WT
	**Av. Age:**	**Gender**	**Race:**	**Ethnicity:**		**Tumor Grade Distribution:**	**Recurrence Distribution:**	**IDH Genotype Distribution:**
	56	M: 24<br>F: 10	W: 30<br>B: 1<br>A.I.: 1<br>Unknown: 2	H: 4<br>N.H.: 28<br>Unknown: 2		Grade I: 0<br>Grade II: 7<br>Grade III: 4<br>Grade IV: 22<br>Unknown: 1	Primary: 25<br>Recurrent: 7<br>Unknown: 2	WT: 23<br>Mut: 11

M= Male; F= Female

W= White; A.I.= Asian Indian; B= Black

H= Hispanic; N.H.= Not Hispanic

P= Primary; R= Recurrent

Mut= Mutant; WT= Wild type

-= *information not provided/available*

***** Necrotic tissue with minimum involvement of oligodendriglioma

Table S5. Biopsy location, MS/MS data, DESI-MS IDH mutation status prediction (Purdue University/Mayo Clinic)

Sample Information				2-HG MS2 Data					DESI-MS Prediction	
**Subject**	**Biopsy # (Subject)**	**Biopsy # overall**	**Location**	**scan #**	***m/z* 128**	***m/z* 129**	**IDH Mutation Score**	**log**	**IDH Mutation Status**	**IDH Coded**
1	1	1	Margin	107	3.005256553	0.442536579	0.0862	-1.0642	Wildtype	0
	3	2	Core	107	2.382997945	0.533537222	0.1628	-0.7880	Wildtype	0
	4	3	Core	107	2.632309814	0.400503918	0.0911	-1.0402	Wildtype	0
	5	4	Core	106	2.097815675	0.374470623	0.1175	-0.9299	Wildtype	0
	6	5	Core	107	4.725273747	0.816321374	0.1117	-0.9517	Wildtype	0
	8	6	Core	107	2.426763444	0.537394221	0.1604	-0.7946	Wildtype	0
2	1	7	Margin	107	1.795691797	0.269959768	0.0893	-1.0490	Wildtype	0
	3	8	Margin	106	1.887280661	0.305710887	0.1010	-0.9957	Wildtype	0
	4	9	Core	107	2.755895691	0.551243801	0.1390	-0.8569	Wildtype	0
	5	10	Core	107	1.912112836	0.513011424	0.2073	-0.6834	Wildtype	0
	6	11	Core	107	3.453631859	0.696519937	0.1407	-0.8518	Wildtype	0
	7	12	Core	107	1.387883681	0.333835326	0.1795	-0.7458	Wildtype	0
	8	13	Core	107	2.986020056	0.506346928	0.1086	-0.9643	Wildtype	0
3	1	14	Core	107	11.67931019	1.223544418	0.0438	-1.3589	Wildtype	0
	2	15	Core	107	15.47535488	1.83024694	0.0573	-1.2421	Wildtype	0
	3	16	Core	107	21.31272942	2.324704519	0.0481	-1.3181	Wildtype	0
	4	17	Core	107	9.903904953	1.350514949	0.0754	-1.1228	Wildtype	0
	5	18	Core	107	7.025992907	0.77360447	0.0491	-1.3089	Wildtype	0
	6	19	Margin	107	26.86627519	2.295936975	0.0245	-1.6116	Wildtype	0
	7	20	Core	107	4.56566036	0.583177726	0.0667	-1.1757	Wildtype	0
	8	21	Core	107	8.908468492	1.210395825	0.0749	-1.1257	Wildtype	0
4	2	22	Core	107	17.72286457	2.947832895	0.1053	-0.9775	Wildtype	0
	3	23	Core	106	1.738532078	0.369715932	0.1517	-0.8191	Wildtype	0
	4	24	Core	107	16.13058214	2.43801055	0.0901	-1.0451	Wildtype	0
	5	25	Core	107	1.557154005	0.282716405	0.1206	-0.9188	Wildtype	0
	6	26	Core	107	16.2731872	2.991377185	0.1228	-0.9107	Wildtype	0
	7	27	Core	106	1.170863106	0.250989864	0.1534	-0.8143	Wildtype	0
	8	28	Core	107	16.07095122	2.212567708	0.0767	-1.1153	Wildtype	0
5	1	29	Margin	107	17.30050719	24.79684604	1.3723	0.1374	Mutant	1

	2	30	Margin	107	17.66502455	17.94200784	0.9547	-0.0201	Wildtype	0
	3	31	Core	107	3.485341408	86.61421603	24.7900	1.3943	Mutant	1
	4	32	Core	107	19.57152504	30.43988126	1.4943	0.1744	Mutant	1
	5	33	Core	107	3.47498415	104.2948365	29.9520	1.4764	Mutant	1
	6	34	Core	107	3.842167492	109.2818162	28.3818	1.4530	Mutant	1
	7	35	Margin	107	14.56463944	18.33664937	1.1980	0.0785	Wildtype	0
	8	36	Margin	107	4.089847505	23.09893992	5.5869	0.7472	Mutant	1
6	1	37	Margin	107	9.965785086	2.01124286	0.1408	-0.8514	Wildtype	0
	2	38	Margin	107	7.590373948	1.575398542	0.1466	-0.8340	Wildtype	0
	3	39	Margin	107	2.382292023	0.469963454	0.1363	-0.8656	Wildtype	0
	4	40	Margin	107	3.710116155	0.807984261	0.1568	-0.8047	Wildtype	0
	5	41	Core	99	0.917132407	0.190085154	0.1463	-0.8349	Wildtype	0
	6	42	Core	106	1.514097714	0.25600697	0.1081	-0.9662	Wildtype	0
	7	43	Core	107	2.598594462	0.362987527	0.0787	-1.1041	Wildtype	0
	8	44	Core	107	12.7955205	1.49769329	0.0560	-1.2514	Wildtype	0
7	1	45	Core	107	7.159538157	1.160019721	0.1010	-0.9956	Wildtype	0
	2	46	Core	107	4.399843059	1.202733382	0.2124	-0.6729	Wildtype	0
	3	47	Core	107	3.759689889	0.564051073	0.0890	-1.0505	Wildtype	0
	4	48	Core	107	6.084544436	1.017033567	0.1062	-0.9741	Wildtype	0
	5	49	Margin	107	13.0043686	1.40602161	0.0471	-1.3268	Wildtype	0
	6	50	Core	106	3.687186588	0.727305068	0.1363	-0.8657	Wildtype	0
	7	51	Core	107	3.618271039	0.727533512	0.1401	-0.8536	Wildtype	0
	8	52	Core	107	4.685843501	0.731405327	0.0951	-1.0219	Wildtype	0
8	1	53	Margin	107	2.432768669	53.092532	21.7629	1.3377	Mutant	1
	2	54	Margin	107	0.878417337	51.96283217	59.0941	1.7715	Mutant	1
	3	55	Margin	106	1.076269557	23.45265939	21.7297	1.3371	Mutant	1
	4	56	Margin	105	0.783421161	41.19789407	52.5262	1.7204	Mutant	1
	5	57	Core	107	2.353237249	49.19266428	20.8433	1.3190	Mutant	1
	6	58	Margin	107	6.385557976	24.6377625	3.7974	0.5795	Mutant	1
	7	59	Margin	105	1.221855386	70.03050107	57.2539	1.7578	Mutant	1
	8	60	Core	107	4.707259171	45.79941898	9.6685	0.9854	Mutant	1
9	1	61	Margin	107	3.677443463	0.450813336	0.0616	-1.2105	Wildtype	0
	2	62	Margin	106	7.876361414	0.684443619	0.0259	-1.5867	Wildtype	0
	3	63	Core	107	8.926896286	0.859236944	0.0353	-1.4528	Wildtype	0

	4	64	Margin	107	16.81381996	1.535273716	0.0303	-1.5184	Wildtype	0
	5	65	Core	107	5.95917995	1.281181179	0.1540	-0.8125	Wildtype	0
	6	66	Core	106	3.321658159	0.501993122	0.0901	-1.0451	Wildtype	0
	8	67	Core	107	5.899600735	0.819725204	0.0779	-1.1082	Wildtype	0
10	1	68	Core	107	12.33247054	1.165543766	0.0335	-1.4748	Wildtype	0
	2	69	Core	107	6.469629736	0.899988847	0.0781	-1.1073	Wildtype	0
	3	70	Margin	107	8.556849491	0.924897107	0.0471	-1.3271	Wildtype	0
	4	71	Core	107	4.623925866	0.68105586	0.0863	-1.0640	Wildtype	0
	5	72	Core	107	2.388093881	0.425058128	0.1170	-0.9318	Wildtype	0
	6	73	Core	95	0.715132106	0.18020175	0.1910	-0.7190	Wildtype	0
	7	74	Core	107	3.836211387	0.670311946	0.1137	-0.9441	Wildtype	0
	8	75	Core	107	5.977749179	0.699743973	0.0561	-1.2514	Wildtype	0
11	1	76	Margin	107	5.439326012	1.011430172	0.1249	-0.9033	Wildtype	0
	2	77	Margin	107	10.51618979	1.493432445	0.0810	-1.0914	Wildtype	0
	3	78	Core	106	2.225635007	0.259148859	0.0554	-1.2562	Wildtype	0
	4	79	Core	92	0.727962742	0.125588244	0.1115	-0.9526	Wildtype	0
	5	80	Core	107	3.198258874	0.473453116	0.0870	-1.0603	Wildtype	0
	6	81	Core	89	0.835699143	0.189614972	0.1659	-0.7802	Wildtype	0
12	1	82	Margin	107	10.23016625	1.220410545	0.0583	-1.2344	Wildtype	0
	2	83	Margin	107	10.53551251	1.430355041	0.0748	-1.1263	Wildtype	0
	3	84	Margin	107	9.004659572	1.020933674	0.0524	-1.2808	Wildtype	0
	5	85	Core	107	3.002495653	0.408814028	0.0752	-1.1240	Wildtype	0
	7	86	Margin	107	10.79143452	1.462808329	0.0746	-1.1275	Wildtype	0
	8	87	Core	107	2.38860444	0.517654353	0.1557	-0.8077	Wildtype	0
13	1	88	Margin	56	0.232064733	5.787460907	24.8780	1.3958	Mutant	1
	2	89	Margin	82	0.338495491	25.77265007	76.0778	1.8813	Mutant	1
	3	90	Core	105	0.973662899	47.37842803	48.5990	1.6866	Mutant	1
	4	91	Core	102	0.976713708	73.03212645	74.7123	1.8734	Mutant	1
	5	92	Core	98	0.702891757	49.06957578	69.7500	1.8435	Mutant	1
	6	93	Core	69	0.22451517	18.97175902	84.4400	1.9265	Mutant	1
	7	94	Core	87	0.29687183	29.73785271	100.1097	2.0005	Mutant	1
	8	95	Core	96	0.766999156	28.08060868	36.5500	1.5629	Mutant	1
14	1	96	Margin	107	1.866626469	14.78332746	7.8588	0.8954	Mutant	1
	2	97	Margin	107	7.622958485	28.37709876	3.6616	0.5637	Mutant	1

	3	98	Margin	107	1.738893223	22.55964183	12.9126	1.1110	Mutant	1
	4	99	Core	106	1.968150729	19.68257428	9.9395	0.9974	Mutant	1
	5	100	Core	106	3.716625041	34.18653936	9.1373	0.9608	Mutant	1
	6	101	Core	107	8.592941301	18.75480467	2.1216	0.3267	Mutant	1
	7	102	Core	107	11.78152872	30.78020378	2.5516	0.4068	Mutant	1
	8	103	Core	107	18.68767892	3.917388461	0.1486	-0.8279	Wildtype	0
15	1	104	Margin	107	3.89396955	49.04234526	12.5334	1.0981	Mutant	1
	2	105	Margin	107	8.238460717	38.73882261	4.6412	0.6666	Mutant	1
	3	106	Margin	107	5.309659776	53.86459573	10.0836	1.0036	Mutant	1
	4	107	Margin	107	4.343553828	50.78637064	11.6314	1.0656	Mutant	1
	5	108	Core	107	7.689287167	51.81485623	6.6776	0.8246	Mutant	1
	6	109	Core	107	6.899139699	46.1606161	6.6298	0.8215	Mutant	1
	7	110	Core	107	9.574599929	40.89282432	4.2100	0.6243	Mutant	1
	8	111	Margin	107	7.029687617	7.341078194	0.9833	-0.0073	Wildtype	0
16	1	112	Margin	107	5.232479788	0.65802936	0.0648	-1.1887	Wildtype	0
	2	113	Margin	107	14.3269937	2.066268091	0.0832	-1.0798	Wildtype	0
	3	114	Margin	107	10.23515972	1.220833049	0.0583	-1.2345	Wildtype	0
	4	115	Core	107	10.23515972	1.220833049	0.0583	-1.2345	Wildtype	0
17	1	116	Margin	107	6.001967793	1.177833025	0.1352	-0.8689	Wildtype	0
	2	117	Margin	107	7.532782765	1.423219835	0.1279	-0.8930	Wildtype	0
	3	118	Margin	107	5.636828343	1.215633788	0.1547	-0.8106	Wildtype	0
	4	119	Margin	105	2.596576204	0.597989582	0.1693	-0.7713	Wildtype	0
	5	120	Core	107	9.772264645	1.508182014	0.0933	-1.0300	Wildtype	0
18	1	121	Margin	107	4.586913854	3.376993425	0.6752	-0.1706	Wildtype	0
	2	122	Margin	105	1.472672237	3.247353803	2.1441	0.3312	Mutant	1
	3	123	Margin	107	5.004409543	1.860110632	0.3107	-0.5077	Wildtype	0
	4	124	Margin	107	14.61651596	2.630520622	0.1190	-0.9246	Wildtype	0
	5	125	Core	107	12.55912156	2.248667547	0.1180	-0.9279	Wildtype	0
	6	126	Core	106	2.087374448	5.886286679	2.7589	0.4407	Mutant	1
	7	127	Core	107	6.951993485	10.15778712	1.4001	0.1462	Mutant	1
	8	128	Core	107	2.294449711	7.514643637	3.2141	0.5071	Mutant	1
19	1	129	Core	107	7.910147802	1.042424107	0.0708	-1.1501	Wildtype	0
	2	130	Core	107	8.346166675	1.060478177	0.0661	-1.1801	Wildtype	0
	5	131	Core	106	4.737523316	0.736239473	0.0944	-1.0250	Wildtype	0

	6	132	Core	107	4.802382974	0.779341493	0.1013	-0.9945	Wildtype	0
	7	133	Core	107	6.962909175	0.79230718	0.0528	-1.2775	Wildtype	0
	8	134	Core	107	7.100651116	0.603100913	0.0239	-1.6209	Wildtype	0
20	2	135	Core	104	5.671316678	0.741963073	0.0698	-1.1560	Wildtype	0
	3	136	Core	105	4.590982123	0.690355037	0.0894	-1.0488	Wildtype	0
	4	137	Core	98	1.554313988	0.318358272	0.1438	-0.8422	Wildtype	0
	5	138	Core	69	0.602826882	0.332544483	0.4906	-0.3092	Wildtype	0
	6	139	Core	90	0.950322111	0.368102721	0.3263	-0.4863	Wildtype	0
	7	140	Core	87	1.209378486	0.251523134	0.1470	-0.8328	Wildtype	0
	8	141	Core	100	2.469950916	0.383396058	0.0942	-1.0258	Wildtype	0
21	1	142	Margin	107	11.5651109	32.46685467	2.7463	0.4387	Mutant	1
	2	143	Margin	106	4.208048154	33.16639206	7.8207	0.8932	Mutant	1
	3	144	Margin	105	1.756191754	7.952404239	4.4672	0.6500	Mutant	1
	4	145	Margin	106	3.582590162	35.40237165	9.8208	0.9921	Mutant	1
	5	146	Margin	107	2.787673932	17.75392032	6.3077	0.7999	Mutant	1
	6	147	Margin	107	3.819137804	13.67305583	3.5191	0.5464	Mutant	1
	7	148	Core	103	1.679533759	10.88188582	6.4181	0.8074	Mutant	1
	8	149	Core	106	3.588433233	15.68335709	4.3095	0.6344	Mutant	1
22	1	150	-	106	11.80321976	1.158089333	0.0371	-1.4304	Wildtype	0
	2	151	-	107	8.582551276	1.384714544	0.1003	-0.9985	Wildtype	0
	3	152	-	107	8.674187828	1.188695456	0.0760	-1.1190	Wildtype	0
	4	153	-	103	3.761454867	0.473105863	0.0648	-1.1886	Wildtype	0
	5	154	-	105	4.190963361	0.449645559	0.0463	-1.3345	Wildtype	0
	6	155	-	107	6.496733064	1.073236044	0.1042	-0.9821	Wildtype	0
	7	156	-	102	6.684282898	1.187713938	0.1167	-0.9330	Wildtype	0
	8	157	-	104	4.081050437	0.888631192	0.1567	-0.8048	Wildtype	0
23	1	158	Margin	107	16.64220289	1.705353174	0.0415	-1.3822	Wildtype	0
	2	159	Margin	107	11.15945878	1.133859298	0.0406	-1.3914	Wildtype	0
	3	160	Core	107	14.57621155	1.481328539	0.0406	-1.3912	Wildtype	0
	4	161	*Margin*	107	5.713929185	0.886152164	0.0941	-1.0265	Wildtype	0
	5	162	Core	107	3.532441345	0.556591073	0.0966	-1.0152	Wildtype	0
	6	163	Core	107	11.08910939	1.20080458	0.0473	-1.3253	Wildtype	0
	7	164	Core	107	6.438569553	0.644133705	0.0390	-1.4085	Wildtype	0
	8	165	Margin	107	9.564920202	1.10206138	0.0542	-1.2658	Wildtype	0

24	1	166	Margin	107	3.650263216	0.530976952	0.0845	-1.0733	Wildtype	0
	2	167	Margin	107	7.158643347	0.770446653	0.0466	-1.3314	Wildtype	0
	3	168	Margin	74	0.480456204	5.648967024	11.6965	1.0681	Mutant	1
	4	169	Core	102	1.533651303	7.405727163	4.7678	0.6783	Mutant	1
	5	170	Margin	91	0.695806885	16.25712876	23.3034	1.3674	Mutant	1
	6	171	Core	63	0.454420144	10.71785336	23.5248	1.3715	Mutant	1
	7	172	Core	73	0.638382461	11.575285	18.0712	1.2570	Mutant	1
	8	173	Core	82	1.214944896	7.982015593	6.5089	0.8135	Mutant	1
25	3	174	-	107	2.644932145	5.703583269	2.0954	0.3213	Mutant	1
	4	175	-	98	0.94182586	6.886215048	7.2506	0.8604	Mutant	1
	5	176	-	103	1.518267835	11.01891922	7.1966	0.8571	Mutant	1
	6	177	-	103	1.48976866	9.627136816	6.4012	0.8063	Mutant	1
	7	178	-	103	1.151879457	7.76097241	6.6767	0.8246	Mutant	1
	8	179	-	107	1.316183077	9.617040941	7.2458	0.8601	Mutant	1
26	1	180	Margin	97	1.103517398	0.256761332	0.1717	-0.7653	Wildtype	0
	2	181	Margin	95	1.77019423	0.192523162	0.0478	-1.3210	Wildtype	0
	3	182	Margin	22	0.378234339	0.089543059	0.1757	-0.7551	Wildtype	0
	4	183	Core	44	0.378349407	0.108912713	0.2269	-0.6442	Wildtype	0
	5	184	Core	100	1.84661283	0.22025978	0.0583	-1.2345	Wildtype	0
	6	185	Core	36	0.476321568	0.099305029	0.1475	-0.8313	Wildtype	0
	7	186	Core	53	0.564905614	0.136292188	0.1803	-0.7441	Wildtype	0
	8	187	Core	99	1.255920261	0.242746024	0.1323	-0.8785	Wildtype	0
27	1	188	-	91	0.706575007	0.208408908	0.2340	-0.6309	Wildtype	0
	2	189	-	59	0.492894072	0.123133249	0.1888	-0.7240	Wildtype	0
	3	190	-	43	0.391769018	0.187389687	0.4173	-0.3795	Wildtype	0
	4	191	-	61	0.594392623	0.155639749	0.2008	-0.6971	Wildtype	0
28	1	192	Margin	107	5.279115624	0.76409391	0.0837	-1.0771	Wildtype	0
	2	193	Margin	98	1.707044369	0.227865388	0.0725	-1.1397	Wildtype	0
	3	194	Margin	102	1.520928	0.246066584	0.1008	-0.9966	Wildtype	0
	4	195	Margin	101	1.284202393	0.190570832	0.0874	-1.0585	Wildtype	0
	6	196	Core	56	0.428590401	0.073306307	0.1100	-0.9584	Wildtype	0
	7	197	Core	98	1.049390243	0.137354658	0.0699	-1.1556	Wildtype	0
	8	198	Core	82	0.62141088	0.146945042	0.1755	-0.7558	Wildtype	0
29	1	199	Core	90	0.811606344	9.899181206	12.1360	1.0841	Mutant	1

	2	200	Core	56	0.339091951	9.09910903	26.7728	1.4277	Mutant	1
	3	201	Margin	97	1.417952276	7.359106618	5.1290	0.7100	Mutant	1
	4	202	Margin	54	0.444554163	7.832060722	17.5568	1.2444	Mutant	1
	5	203	Margin	28	0.266257609	3.469368312	12.9691	1.1129	Mutant	1
	6	204	Margin	53	0.370701661	7.715429351	20.7520	1.3171	Mutant	1
	7	205	Margin	78	0.649483929	12.2968622	18.8723	1.2758	Mutant	1
	8	206	Core	67	0.690341927	5.262176557	7.5616	0.8786	Mutant	1
30	1	207	Margin	61	0.44543155	0.132969246	0.2375	-0.6243	Wildtype	0
	2	208	Core	52	0.509190408	0.136437469	0.2069	-0.6841	Wildtype	0
	3	209	core	20	0.26450652	1.56529E-05	0.00006	-4.2218	Wildtype	0
	4	210	Margin	81	0.68171255	0.186960322	0.2132	-0.6711	Wildtype	0
	5	211	Core	75	0.658989496	0.11516155	0.1137	-0.9440	Wildtype	0
	6	212	Core	83	0.857862923	0.173667366	0.1414	-0.8494	Wildtype	0
	7	213	Core	91	0.694255694	0.14743753	0.1513	-0.81996	Wildtype	0
	8	214	Core	95	1.541497714	0.326868934	0.1510	-0.8208	Wildtype	0
31	4	215	Margin	92	1.119253095	0.183588943	0.1030	-0.9870	Wildtype	0
	5	216	Margin	102	3.142982756	0.317049597	0.0399	-1.3993	Wildtype	0
	6	217	Core	89	0.603880567	0.116374903	0.1317	-0.8804	Wildtype	0
	7	218	Core	84	0.950471214	0.191182624	0.1401	-0.8534	Wildtype	0
	8	219	Core	104	1.246198822	0.236419893	0.1287	-0.8904	Wildtype	0
32	1	220	Margin	100	8.107538067	0.558491482	0.0079	-2.1032	Wildtype	0
	2	221	Margin	103	1.925323002	0.250289776	0.0690	-1.1612	Wildtype	0
	3	222	Core	104	1.261636948	0.204095594	0.1008	-0.9967	Wildtype	0
	4	223	Margin	107	4.042906475	1.083462898	0.2070	-0.6840	Wildtype	0
	5	224	Core	107	3.702700507	1.038217798	0.2194	-0.6588	Wildtype	0
	6	225	Core	103	1.438385992	0.371702589	0.1974	-0.7046	Wildtype	0
	7	226	Core	103	1.664266949	0.299711013	0.1191	-0.9241	Wildtype	0
	8	227	Margin	102	1.426451232	0.610302196	0.3668	-0.4355	Wildtype	0
33	1	228	Margin	101	1.76311974	9.073017086	5.0850	0.7063	Mutant	1
	2	229	Core	107	4.333037345	6.930848065	1.5385	0.1871	Mutant	1
	5	230	Margin	104	1.856554288	0.254769883	0.0762	-1.1179	Wildtype	0
	6	231	Margin	104	3.023671419	0.373634828	0.0626	-1.2036	Wildtype	0
	7	232	Core	99	0.92542383	6.392010303	6.8461	0.8354	Mutant	1
34	1	233	Margin	107	2.410493171	0.268807353	0.0505	-1.2966	Wildtype	0

	2	234	Margin	107	2.821081353	0.382839216	0.0747	-1.1266	Wildtype	0
	3	235	Margin	107	3.189660916	0.387450144	0.0605	-1.2185	Wildtype	0
	4	236	Margin	95	0.958261875	0.124876128	0.0693	-1.1592	Wildtype	0
	5	237	Core	104	1.978247944	0.261949527	0.0714	-1.1462	Wildtype	0
	6	238	Core	107	3.901064151	0.401166754	0.0418	-1.3785	Wildtype	0
	7	239	Core	107	2.954469015	0.292242032	0.0379	-1.4212	Wildtype	0
	8	240	Core	107	3.791430967	0.464484894	0.0615	-1.2111	Wildtype	0

Table S6. Clinical demographics, tumor diagnosis, Miniature MS results for patients in the training data set (Tsinghua University/Huashan Hospital study).

Subject #	Gender	Age	Diagnosis	Grade	biopsy # (subject)	biopsy # (overall)	Tumor /Normal	IHC	PCR	MS2 2-HG/GLU	Rel. Int. of 2-HG	Rel. Int. of GLU	Rel. Int. of NAA	Rel. Int. of m/z = 129
1	Male	16	Glioblastoma	4	1	1	Tumor tissue	-	Wild -type	0.032532	0.058975	0.403427	0.027034	0.008666
2	Female	32	Astrocytoma	2	1	2	Tumor tissue	+	R132 H	4,993.11	0.344223	0.015001	0.022824	0.058853
3	Female	44	Glioblastoma	4	1	3	Tumor tissue	-	Wild -type	0.013659	0.072514	0.439479	0.101085	0.008635
4	Male	60	Glioblastoma	4	1	4	Tumor tissue	-	Wild -type	0.010095	0.043741	0.517836	0.078635	0.011209
5	Male	66	Glioblastoma	4	1	5	Tumor tissue	-	Wild -type	0.022801	0.081311	0.407168	0.020052	0.007969
6	Female	56	Glioblastoma	4	1	6	Tumor tissue	-	Wild -type	0.047928	0.055622	0.572362	0.081568	0.006930
	Female	58	Astrocytoma	2	1	7	Tumor tissue	-	Wild -type	0.026387	0.052254	0.387108	0.217200	0.049322
7	Female	58	Astrocytoma	2	2	8	Tumor tissue	-	Wild -type	0.001088	0.080612	0.384947	0.177999	0.010188
	Female	58	Astrocytoma	2	3	9	Tumor tissue	-	Wild -type	0.058232	0.064104	0.406669	0.132577	0.007544
8	Male	55	Astrocytoma	3	1	10	Tumor tissue	-	Wild -type	0.020219	0.051667	0.569099	0.036210	0.029060
9	Male	52	Oligodendroglioma	2	1	11	Tumor tissue	+	R132 H	0.394870	0.220713	0.243571	0.201284	0.031387
	Male	52	Oligodendroglioma	2	2	12	Tumor tissue	+	R132 H	0.753155	0.292489	0.318576	0.035472	0.067961
10	Male	30	Astrocytoma	4	1	13	Tumor tissue	+	R132 H	47.299710	0.687323	0.015759	0.089362	0.055097
11	Female	28	Glioblastoma	4	1	14	Tumor tissue	-	Wild -type	0.028166	0.029830	0.252209	0.000441	0.043704
12	Female	25	Astrocytoma	2	1	15	Tumor tissue	-	Wild -type	0.052692	0.040871	0.548482	0.140024	0.003520
	Female	25	Astrocytoma	2	2	16	Tumor tissue	-	Wild -type	0.055798	0.048693	0.591801	0.025427	0.012140
13	Male	34	Glioblastoma	4	1	17	Tumor tissue	-	Wild -type	0.020359	0.028922	0.350318	0.023847	0.026887

14	Male	60	Glioblastoma	4	1	18	Tumor tissue	-	Wild-type	0.005624	0.071094	0.584616	0.040118	0.017036
15	Male	45	Astrocytoma	3	1	19	Tumor tissue	-	Wild-type	0.037581	0.063800	0.652755	0.064630	0.006483
16	Male	42	Astrocytoma	2	1	20	Tumor tissue	+	R132H	19.636468	0.544097	0.006613	0.039971	0.024223
	Male	42	Astrocytoma	2	2	21	Tumor tissue	+	R132H	3.643448	0.411217	0.102127	0.026126	0.050609
17	Female	35	Oligodendroglioma	3	1	22	Tumor tissue	+	R132H	10.321549	0.721678	0.047052	0.013200	0.179382
18	Female	50	Oligodendroglioma	3	1	23	Tumor tissue	+	R132H	0.396415	0.131512	0.360236	0.063047	0.010427
19	Female	28	Oligodendroglioma	3	1	24	Tumor tissue	+-	R132H	2.592080	0.470850	0.233344	0.006311	0.075695
20	Female	69	Astrocytoma	3	1	25	Tumor tissue	-	Wild-type	0.068990	0.101910	0.417118	0.018690	0.014824
21	Male	70	Glioblastoma	4	1	26	Tumor tissue	-+	Wild-type	0.029019	0.101762	0.494515	0.069837	0.021738
	Male	70	Glioblastoma	4	2	27	Tumor tissue	-+	Wild-type	0.011449	0.060109	0.456486	0.017365	0.000383
22	Female	34	Astrocytoma	2	3	28	Tumor tissue	+	R132H	1.431999	0.383883	0.180431	0.022191	0.031093
23	Male	44	Astrocytoma	2	1	29	Tumor tissue	-	R132H	1.053259	0.392549	0.205199	0.243653	0.032514
24	Male	66	Glioblastoma	4	1	30	Tumor tissue	-	Wild-type	0.025994	0.042012	0.598634	0.012988	0.020949
25	Male	80	Glioblastoma	4	1	31	Tumor tissue	-	Wild-type	0.060126	0.076650	0.484994	0.037006	0.012671
26	Female	30	Astrocytoma	2	1	32	Tumor tissue	-	Wild-type	4.765639	0.480631	0.156415	0.105852	0.065681
27	Male	22	Astrocytoma	3	1	33	Tumor tissue	+	R132H	0.316466	0.137541	0.373529	0.061655	0.024739
28	Male	47	Glioblastoma	4	1	34	Tumor tissue	-	Wild-type	0.052042	0.077284	0.270167	0.004040	0.010883
29	Male	64	Glioblastoma	4	1	35	Tumor tissue	-	Wild-type	0.082484	0.196745	0.097207	0.029891	0.015060
30	Female	47	Glioblastoma	4	1	36	Tumor tissue	-	Wild-type	0.055024	0.086625	0.096789	0.075154	0.024696

31	Male	50	Glioblastoma	4	1	37	Tumor tissue	-	Wild -type	0.009795	0.049704	0.507630	0.008942	0.024336
32	Female	23	Astrocytoma	2	1	38	Tumor tissue	-	Wild -type	0.012310	0.027184	0.451641	0.193413	0.003233
33	Male	47	Glioblastoma	4	1	39	Tumor tissue	-	Wild -type	0.028517	0.070832	0.423135	0.014676	0.009719
34	Male	45	Glioblastoma	4	1	40	Tumor tissue	-	Wild -type	0.059121	0.061951	0.468064	0.032920	0.022954
35	Male	43	Astrocytoma	2	1	41	Tumor tissue	+	R132 H	86.007798	0.492947	0.006557	0.019042	0.047015
36	Female	53	Astrocytoma	2	1	42	Tumor tissue	-	R132 H	172.22045	0.158404	0.000052	0.002417	0.080099
37	Female	32	Astrocytoma	3	1	43	Tumor tissue	+	R132 H	0.655604	0.263387	0.370037	0.027381	0.009041
38	Male	68	Glioblastoma	4	1	44	Tumor tissue	-	Wild -type	0.017727	0.063555	0.525104	0.045138	0.009867
39	Female	65	Glioblastoma	4	1	45	Tumor tissue	-	Wild -type	0.015280	0.054409	0.477602	0.022434	0.023921
40	Male	55	Glioblastoma	4	1	46	Tumor tissue	-	Wild -type	1.372158	0.340912	0.284450	0.018343	0.015537
41	Female	47	Pilocyticastrocytoma	1	1	47	Tumor tissue	-	Wild -type	0.039079	0.037064	0.517121	0.086636	0.008061
42	Male	24	Astrocytoma	2	1	48	Tumor tissue	-	Wild -type	0.005162	0.088617	0.309946	0.046627	0.015980
43	Male	42	Glioblastoma	4	1	49	Tumor tissue	-	Wild -type	0.064406	0.057254	0.316079	0.010423	0.007612
44	Male	59	Glioblastoma	4	1	50	Tumor tissue	-	Wild -type	0.046014	0.050476	0.429663	0.003296	0.027473
45	Male	36	Oligodendroglioma	2	1	51	Tumor tissue	-+	R132 H	3.728978	0.483828	0.154526	0.008924	0.088039
46	Male	33	Astrocytoma	2	1	52	Tumor tissue	+-	Wild -type	0.057663	0.080846	0.489058	0.071615	0.003495
	Male	33	Astrocytoma	2	2	53	Tumor tissue	+-	Wild -type	0.047753	0.018153	0.275592	0.325409	0.009891
47	Female	63	Glioblastoma	4	1	54	Tumor tissue	-	Wild -type	0.029867	0.041650	0.235445	0.017036	0.011302
48	Male	15	Glioblastoma	4	1	55	Tumor tissue	-	Wild -type	0.134551	0.039588	0.462301	0.084808	0.034325

49	Male	65	Glioblastoma	4	1	56	Tumor tissue	-	Wild -type	0.015474	0.053755	0.444236	0.058512	0.018605
50	Male	41	Astrocytoma	2	1	57	Tumor tissue	-	Wild -type	0.005095	0.057907	0.433933	0.016361	0.008384
51	Female	60	Glioblastoma	4	1	58	Tumor tissue	-	Wild -type	0.028549	0.108432	0.260790	0.015917	0.019230
52	Female	60	Astrocytoma	3	1	59	Tumor tissue	-	Wild -type	0.051289	0.064061	0.433952	0.018488	0.012841
53	Male	53	Astrocytoma	3	1	60	Tumor tissue	-	Wild -type	0.115184	0.039006	0.396098	0.060891	0.059534
54	Female	55	Oligodendroglioma	2	1	61	Tumor tissue	+	R132H	51.637508	0.560125	0.016705	0.014449	0.032706
55	Female	50	Glioblastoma	4	1	62	Tumor tissue	-	Wild -type	0.102627	0.047018	0.228169	0.053466	0.014597
56	Female	32	Astrocytoma	4	1	63	Tumor tissue	+	R132H	1.660080	0.385368	0.208809	0.039484	0.037306
57	Male	47	Oligodendroglioma	3	1	64	Tumor tissue	+	R132H	26.105141	0.424082	0.041727	0.030050	0.103182
58	Male	47	Astrocytoma	4	1	65	Tumor tissue	+	R132H	0.482364	0.179119	0.408899	0.255114	0.014144
59	Female	45	Oligodendroglioma	2	1	66	Tumor tissue	+	R132H	0.831823	0.248407	0.322631	0.071496	0.027472
	Male	36	Astrocytoma	2	1	67	Tumor tissue	+	R132H	0.483927	0.235415	0.289564	0.028525	0.024726
60	Male	36	Astrocytoma	2	2	68	Tumor tissue	+	R132H	0.800073	0.302781	0.281056	0.003337	0.022910
	Male	36	Astrocytoma	2	3	69	Tumor tissue	+	R132H	1.001368	0.345931	0.378926	0.026086	0.052499
61	Male	48	Astrocytoma	2	1	70	Tumor tissue	-	R172K	33.739258	0.605660	0.028170	0.105681	0.149087
62	Female	22	Pilocyticastrocytoma	1	1	71	Tumor tissue	-	Wild -type	0.143048	0.107115	0.305238	0.129564	0.035775
63	Female	34	Astrocytoma	2	1	72	Tumor tissue	+	R132H	0.735645	0.268860	0.212888	0.031611	0.018446
	Female	34	Astrocytoma	2	2	73	Tumor tissue	+	R132H	0.339026	0.122492	0.403146	0.047111	0.007430
64	Male	56	Glioblastoma	4	1	74	Tumor tissue	-	Wild -type	0.019198	0.065633	0.361784	0.029124	0.027633

65	Male	25	Astrocytoma	2	1	75	Tumor tissue	-	R172 K	1.726698	0.416350	0.317338	0.376204	0.035856
66	Female	34	Astrocytoma	4	1	76	Tumor tissue	+	R132 H	2.907935	0.446632	0.224085	0.148997	0.047493
	Female	34	Astrocytoma	4	2	77	Tumor tissue	+	R132 H	2.563834	0.446240	0.217523	0.139223	0.040703
67	Female	32	Oligodendroglioma	3	1	78	Tumor tissue	+	R132 H	1.946250	0.430458	0.185120	0.020946	0.024680
68	Male	61	Glioblastoma	4	1	79	Tumor tissue	-	Wild-type	0.018856	0.065001	0.254218	0.057284	0.023832
69	Female	77	Glioblastoma	4	1	80	Tumor tissue	-	Wild-type	0.032126	0.118841	0.235084	0.048382	0.016035
70	Male	38	Glioblastoma	4	1	81	Tumor tissue	-	Wild-type	0.045147	0.035147	0.340383	0.044494	0.009352
71	Male	50	Oligodendroglioma	2	1	82	Tumor tissue	+	R132 H	0.753690	0.234313	0.329787	0.040556	0.037286
	Male	50	Oligodendroglioma	2	2	83	Tumor tissue	+	R132 H	4.409327	0.524483	0.115270	0.011122	0.041788
72	Female	43	Astrocytoma	2	1	84	Tumor tissue	+	R132 H	64.060766	0.192029	0.001668	0.015817	0.067794
73	Male	48	Glioblastoma	4	1	85	Tumor tissue	-	Wild-type	0.067550	0.067212	0.365682	0.044283	0.071609
74	Male	59	Oligodendroglioma	3	1	86	Tumor tissue	+	R132 H	1.206618	0.355324	0.241193	0.009729	0.027469
	Male	57	Oligodendroglioma	2	2	87	Tumor tissue	+	R132 H	6.130217	0.345261	0.055297	0.014828	0.094311
75	Male	53	Oligodendroglioma	2	1	88	Tumor tissue	+	R132 H	1.398049	0.323611	0.279567	0.051352	0.036520
76	Male	43	Oligodendroglioma	2	1	89	Tumor tissue	-	R132 H	1.693299	0.385057	0.228509	0.015355	0.058417
77	Female	74	Glioblastoma	4	1	90	Tumor tissue	-	Wild-type	0.046688	0.051730	0.398678	0.030107	0.018780
78	Male	41	Astrocytoma	2	1	91	Tumor tissue	-	R132 H	2.294637	0.450322	0.202908	0.005194	0.035959
79	Male	44	Oligodendroglioma	3	1	92	Tumor tissue	-+	R132 H	1.060243	0.320894	0.299670	0.429020	0.035901
80	Male	45	Glioblastoma	4	1	93	Tumor tissue	-	Wild-type	0.001318	0.065898	0.213716	0.018414	0.081468

81	Female	33	Glioblastoma	4	1	94	Tumor tissue	-	Wild -type	0.056623	0.074255	0.356908	0.033608	0.003648
82	Male	62	Glioblastoma	4	1	95	Tumor tissue	-	Wild -type	0.024948	0.053682	0.325531	0.027432	0.017924
	Female	20	Glioblastoma	4	1	96	Tumor tissue	-	Wild -type	0.084377	0.047585	0.195830	0.218868	0.004428
83	Female	20	Glioblastoma	4	2	97	Tumor tissue	-	Wild -type	0.017213	0.041414	0.506886	0.209454	0.007240
	Female	20	Glioblastoma	4	3	98	Tumor tissue	-	Wild -type	0.007562	0.031392	0.462019	0.191343	0.008680
84	Male	15	Astrocytoma	3	1	99	Tumor tissue	-	Wild -type	0.056228	0.159479	0.224444	0.007374	0.045102
85	Male	33	Glioblastoma	4	1	100	Tumor tissue	-	Wild -type	0.037452	0.053595	0.482716	0.018526	0.001502
86	Female	38	Oligodendrogli oma	2	1	101	Tumor tissue	+	R132 H	6.533091	0.356020	0.037483	0.231097	0.011604
87	Female	54	Oligodendrogli oma	3	1	102	Tumor tissue	+	R132 H	1.905103	0.399922	0.195043	0.006521	0.075202
88	Male	36	Oligodendrogli oma	3	1	103	Tumor tissue	+	R132 H	1.000313	0.337667	0.316469	0.040155	0.018425
89	Female	44	Astrocytoma	3	1	104	Tumor tissue	-+	R132 H	0.644069	0.334524	0.311916	0.010511	0.055242
90	Male	54	Glioblastoma	4	1	105	Tumor tissue	-	Wild -type	0.013466	0.073160	0.331103	0.028776	0.004704
91	Male	51	Astrocytoma	3	1	106	Tumor tissue	+	R132 H	35.762701	0.487212	0.023906	0.044890	0.044150
92	Female	26	Oligodendrogli oma	2	1	107	Tumor tissue	+	R132 H	0.397261	0.183829	0.308567	0.021013	0.015891
93	Male	37	Oligodendrogli oma	2	1	108	Tumor tissue	+	R132 H	5.948164	0.464152	0.113663	0.025730	0.046304
94	Male	39	Glioblastoma	4	1	109	Tumor tissue	-	Wild -type	0.034154	0.072952	0.431419	0.215412	0.019623
95	Male	50	Glioblastoma	4	1	110	Tumor tissue	-	Wild -type	0.050526	0.048785	0.168214	0.118088	0.031189
96	Male	39	Glioblastoma	4	1	111	Tumor tissue	-	Wild -type	0.057198	0.049572	0.403453	0.016744	0.008770
97	Male	68	Glioblastoma	4	1	112	Tumor tissue	-	Wild -type	0.039818	0.033001	0.455866	0.000157	0.023315

	Male	68	Glioblastoma	4	2	113	Tumor tissue	-	Wild-type	0.044562	0.056659	0.406277	0.048826	0.016122
98	Male	50	Astrocytoma	2	1	114	Tumor tissue	-+	R132H	0.227236	0.140299	0.417355	0.023379	0.032477
	Female	61	Glioblastoma	4	1	115	Tumor tissue	-	Wild-type	0.016397	0.038689	0.506419	0.048791	0.022873
	Female	61	Glioblastoma	4	2	116	Tumor tissue	-	Wild-type	0.065623	0.010554	0.190722	0.030760	0.022251
99	Female	61	Glioblastoma	4	3	117	Tumor tissue	-	Wild-type	0.041779	0.052819	0.507924	0.243792	0.003767
	Female	61	Glioblastoma	4	4	118	Tumor tissue	-	Wild-type	0.048826	0.025241	0.510321	0.010034	0.013803
	Female	61	Glioblastoma	4	5	119	Para-tumor brain tissue	-	Wild-type	0.010984	0.042484	0.648639	0.024018	0.009974
100	Male	47	Oligodendroglioma	3	1	120	Tumor tissue	-	R132H	1.537372	0.347038	0.256873	0.021437	0.035378
101	Male	64	Glioblastoma	4	1	121	Tumor tissue	-	Wild-type	0.005107	0.055627	0.448262	0.104003	0.013198
102	Female	69	Glioblastoma	4	1	122	Tumor tissue	-	Wild-type	0.071967	0.132933	0.169038	0.009644	0.009007
103	Male	64	Astrocytoma	3	1	123	Tumor tissue	-	Wild-type	0.040487	0.031457	0.484636	0.146805	0.015931
104	Male	42	Glioblastoma	4	1	124	Tumor tissue	-	Wild-type	0.031651	0.032421	0.148952	0.047420	0.005438
105	Female	56	Meningioma	1	1	125	Normal brain tissue	-	Wild-type	0.036462	0.080529	0.394325	0.206748	0.024949
	Female	35	Meningioma	1	1	126	Normal brain tissue	-	Wild-type	0.002029	0.050156	0.507705	0.505722	0.014187
106	Female	35	Meningioma	1	2	127	Normal brain tissue	-	Wild-type	0.062777	0.023756	0.229951	0.176931	0.013641
	Female	35	Meningioma	1	3	128	Normal brain tissue	-	Wild-type	0.036423	0.035441	0.436988	0.611809	0.005361
	Female	35	Meningioma	1	4	129	Normal brain tissue	-	Wild-type	0.021023	0.149811	0.326351	0.019551	0.004159
107	Female	56	Neurilemmoma	1	1	130	Normal brain tissue	-	Wild-type	0.049634	0.029201	0.544846	0.397111	0.031457
	Female	56	Neurilemmoma	1	2	131	Normal brain tissue	-	Wild-type	0.013084	0.050012	0.602579	0.548174	0.031566

	Female	56	Neurilemmoma	1	3	132	Normal brain tissue	-	Wild-type	0.049634	0.029201	0.544846	0.397111	0.031457
	Male	57	Astrocytoma	2	1	133	Normal brain tissue	-	Wild-type	0.064329	0.030797	0.570032	0.502226	0.016251
108	Male	57	Astrocytoma	2	2	134	Para-tumor brain tissue	-	Wild-type	0.051533	0.008213	0.316041	0.256382	0.011104
	Male	57	Astrocytoma	2	3	135	Para-tumor brain tissue	-	Wild-type	0.040377	0.028729	0.488438	0.213266	0.017718
109	Female	53	Meningioma	1	1	136	Normal brain tissue	-	Wild-type	0.007333	0.051009	0.542453	0.048286	0.004288
	Female	53	Meningioma	1	2	137	Normal brain tissue	-	Wild-type	0.000366	0.023745	0.516205	0.202758	0.024841

Table S7. Cutoff, sensitivity, specificity, Youden index and AUC of metabolites for the diagnosis of IDH mutation in the training cohort comparing different methods of MS discrimination (Tsinghua University/Huashan Hospital study).

Variables	Cutoff	Sensitivity	Specificity	Youden	AUC	95% Cl	p value
MS^2 of 2HG/Glu	> 0.185	1	0.973	0.973	0.985	0.963- 1.000	<0.001
MS^1 of 2HG	> 0.095	1	0.919	0.919	0.979	0.954- 1.000	<0.001
MS^2 of 2HG/NAA	> 5.645	0.755	0.878	0.634	0.839	0.765- 0.911	<0.001
MS^1 of Glu	< 0.382	0.939	0.608	0.547	0.837	0.769- 0.905	<0.001
MS^1 of *m/z* 129	> 0.024	0.816	0.784	0.600	0.836	0.763- 0.909	<0.001
MS^1 of NAA	< 0.042	0.674	0.500	0.174	0.565	0.460- 0.671	0.221

Table S8 Characteristics of patients in two cohorts (Tsinghua University/Huashan Hospital study)

	Number and (%) of glioma patients [a]		p value
	Training cohort (n = 104)	Validation cohort (n = 70)	
**Age** (year)			0.579[#]
Median (range)	47 (34 - 58)	49 (33 - 60)	
**Sex**			0.342[&]
Male	64 (61.5)	48 (68.6)	
Female	40 (38.5)	22 (31.4)	
**Grade**			0.220[&]
1	2 (1.9)	3 (4.3)	
2	30 (28.8)	26 (37.1)	
3	21 (20.2)	7 (10.0)	
4	51 (49.0)	34 (48.6)	
**IDH status**			0.939[&]
Mutant	41 (39.4)	28 (40.0)	
Wild type	63 (60.6)	42 (60.0)	

Table S9. Cutoff, sensitivity, specificity, and Youden Index of 2-HG/GLU ratio for the detection of IDH mutation in validation set (Tsinghua University/Huashan Hospital study)

Cutoff	Sensitivity	95% CI	Specificity	95% CI	Likelihood Ratio	Youden Index
> 0.001203	1	0.9273 to 1.000	0.01351	0.0006932 to 0.07265	1.014	0.01351
> 0.02944	1	0.9273 to 1.000	0.4054	0.3009 to 0.5192	1.682	0.4054
> 0.07723	1	0.9273 to 1.000	0.8919	0.8009 to 0.9442	9.25	0.8919
> 0.08343	1	0.9273 to 1.000	0.9054	0.8174 to 0.9534	10.57	0.9054
> 0.09350	1	0.9273 to 1.000	0.9189	0.8342 to 0.9623	12.33	0.9189
> 0.1089	1	0.9273 to 1.000	0.9324	0.8514 to 0.9708	14.8	0.9324
> 0.1249	1	0.9273 to 1.000	0.9459	0.8691 to 0.9788	18.5	0.9459
> 0.1388	1	0.9273 to 1.000	0.9595	0.8875 to 0.9890	24.67	0.9595
**> 0.1851**	**1**	**0.9273 to 1.000**	**0.973**	**0.9067 to 0.9952**	**37**	**0.973**
> 0.2719	0.9796	0.8931 to 0.9990	0.973	0.9067 to 0.9952	36.24	0.9526
> 0.3277	0.9592	0.8629 to 0.9927	0.973	0.9067 to 0.9952	35.49	0.9322
> 0.3968	0.898	0.7824 to 0.9556	0.973	0.9067 to 0.9952	33.22	0.871
> 0.5640	0.8367	0.7096 to 0.9149	0.973	0.9067 to 0.9952	30.96	0.8097
> 0.7769	0.7347	0.5974 to 0.8379	0.973	0.9067 to 0.9952	27.18	0.7077
> 0.9161	0.6939	0.5547 to 0.8048	0.973	0.9067 to 0.9952	25.67	0.6669
> 3.686	0.3265	0.2121 to 0.4662	0.9865	0.9273 to 0.9993	24.16	0.313
> 8.427	0.2245	0.1302 to 0.3588	1	0.9507 to 1.000	NA	0.2245

Table S10. Clinical demographics, tumor diagnosis, and Miniature MS results for patients in the validation data set (Tsinghua University/Huashan Hospital study).

Subject #	Gender	Age	Diagnosis	Grade	biopsy # (subject)	biopsy # (overall)	Tumor/Normal Brain Tissue	IHC	PCRseq	2-HG/GLU from Mini-MS	IDH Prediction from Mini-MS
1	Male	86	Glioblastoma	4	1	1	Tumor tissue	-	Wild-type	0.046735277	Wild-type
	Male	86	Glioblastoma	4	2	2	Tumor tissue	-	Wild-type	0.007979802	Wild-type
2	Female	58	Glioblastoma	4	1	3	Tumor tissue	-	Wild-type	0.04189523	Wild-type
	Female	58	Glioblastoma	4	2	4	Tumor tissue	-	Wild-type	0.009146819	Wild-type
	Female	58	Glioblastoma	4	3	5	Tumor tissue	-	Wild-type	0.056651492	Wild-type
3	Male	51	Astrocytoma	2	1	6	Tumor tissue	+	R132H	0.531391304	IDH mutation
4	Male	63	Glioblastoma	4	1	7	Tumor tissue	-	Wild-type	0.01536107	Wild-type
	Male	63	Glioblastoma	4	2	8	Tumor tissue	-	Wild-type	0.041682307	Wild-type
5	Male	64	Oligodendroglioma	3	1	9	Tumor tissue	+	R132H	1.266930175	IDH mutation
	Male	64	Oligodendroglioma	3	2	10	Tumor tissue	+	R132H	1.362913043	IDH mutation
6	Female	20	Glioblastoma	4	1	11	Tumor tissue	-	Wild-type	0.004831279	Wild-type
7	Male	31	Astrocytoma	2	1	12	Tumor tissue	+	R132H	1.397937515	IDH mutation
8	Male	29	Oligodendroglioma	2	1	13	Tumor tissue	+	R132H	1.919075185	IDH mutation
9	Male	50	Oligodendroglioma	2	1	14	Tumor tissue	+	R132H	4.163820703	IDH mutation
	Male	50	Oligodendroglioma	2	2	15	Tumor tissue	+	R132H	13.05258653	IDH mutation
10	Male	73	Glioblastoma	4	1	16	Tumor tissue	-	Wild-type	0.080731404	Wild-type
	Male	73	Glioblastoma	4	2	17	Tumor tissue	-	Wild-type	0.014769408	Wild-type
	Male	73	Glioblastoma	4	3	18	Tumor tissue	-	Wild-type	0.004999862	Wild-type
11	Female	24	Astrocytoma	2	1	19	Tumor tissue	-	Wild-type	0.000852187	Wild-type
12	Female	32	Oligodendroglioma	2	1	20	Tumor tissue	+	R132H	6.534133361	IDH mutation
	Female	32	Oligodendroglioma	2	2	21	Tumor tissue	+	R132H	22.74924089	IDH mutation
	Female	32	Oligodendroglioma	2	3	22	Tumor tissue	+	R132H	1.531122986	IDH mutation
	Female	32	Oligodendroglioma	2	4	23	Tumor tissue	+	R132H	1.637761863	IDH mutation
	Female	32	Oligodendroglioma	2	5	24	Tumor tissue	+	R132H	6.004155122	IDH mutation

	Female	32	Oligodendroglioma	2	6	25	Tumor tissue	+	R132H	2.695101208	IDH mutation
	Female	32	Oligodendroglioma	2	7	26	Para-tumor brain tissue	-	Wild-type	0.052511942	Wild-type
	Female	32	Oligodendroglioma	2	8	27	Para-tumor brain tissue	-	Wild-type	0.037016296	Wild-type
13	Male	34	Oligodendroglioma	2	1	28	Tumor tissue	+	R132H	0.314080202	IDH mutation
	Male	52	Oligodendroglioma	2	1	29	Tumor tissue	+	R132H	5.018014194	IDH mutation
14	Male	52	Oligodendroglioma	2	2	30	Tumor tissue	+	R132H	4.920613972	IDH mutation
	Male	52	Oligodendroglioma	2	3	31	Tumor tissue	+	R132H	0.743358802	IDH mutation
15	Female	62	Glioblastoma	4	1	32	Tumor tissue	-+	Wild-type	0.037016296	Wild-type
	Female	62	Glioblastoma	4	2	33	Tumor tissue	-+	Wild-type	0.052511942	Wild-type
16	Male	54	Glioblastoma	4	1	34	Tumor tissue	-	Wild-type	0.004756876	Wild-type
17	Male	65	Glioblastoma	4	1	35	Tumor tissue	-	Wild-type	0.00175782	Wild-type
	Male	65	Glioblastoma	4	2	36	Tumor tissue	-	Wild-type	0.072485287	Wild-type
	Male	33	Astrocytoma	2	1	37	Tumor tissue	+-	Wild-type	0.094233434	Wild-type
18	Male	33	Astrocytoma	2	2	38	Tumor tissue	+-	Wild-type	0.002164879	Wild-type
	Male	33	Astrocytoma	2	3	39	Tumor tissue	+-	Wild-type	0.038207113	Wild-type
19	Male	36	Astrocytoma	2	1	40	Tumor tissue	+	R132H	4.976351193	IDH mutation
20	Female	34	Astrocytoma	3	1	41	Tumor tissue	+	R132H	1.335734604	IDH mutation
	Female	34	Astrocytoma	3	2	42	Tumor tissue	+	R132H	10.26855797	IDH mutation
21	Female	58	Astrocytoma	2	1	43	Tumor tissue	-	Wild-type	0.09511762	Wild-type
	Female	58	Astrocytoma	2	2	44	Tumor tissue	-	Wild-type	0.0033643	Wild-type
22	Female	75	Glioblastoma	4	1	45	Tumor tissue	-	Wild-type	0.003791957	Wild-type
23	Male	74	Glioblastoma	4	1	46	Tumor tissue	-	Wild-type	0.064022869	Wild-type
	Male	74	Glioblastoma	4	2	47	Tumor tissue	-	Wild-type	0.174181857	Wild-type
	Male	31	Astrocytoma	2	1	48	Tumor tissue	-	R172K	3.710543239	IDH mutation
24	Male	31	Astrocytoma	2	2	49	Tumor tissue	-	R172K	7.013913033	IDH mutation
	Male	31	Astrocytoma	2	3	50	Tumor tissue	-	R172K	6.440174278	IDH mutation
25	Male	28	Glioblastoma	4	1	51	Tumor tissue	-+	Wild-type	0.028778766	Wild-type
26	Male	59	Oligodendroglioma	2	1	52	Tumor tissue	+	R132H	0.548773862	IDH mutation
	Male	59	Oligodendroglioma	2	2	53	Tumor tissue	+	R132H	0.511784883	IDH mutation

	Male	59	Oligodendroglioma	2	3	54	Tumor tissue	+	R132H	6.722552936	IDH mutation
	Male	59	Oligodendroglioma	2	4	55	Tumor tissue	+	R132H	1.067660481	IDH mutation
	Male	59	Oligodendroglioma	2	5	56	Tumor tissue	+	R132H	1.366758754	IDH mutation
	Male	59	Oligodendroglioma	2	6	57	Para-tumor brain tissue	-	Wild-type	0.081380238	Wild-type
	Male	59	Oligodendroglioma	2	7	58	Para-tumor brain tissue	-	Wild-type	0.040290193	Wild-type
	Male	59	Oligodendroglioma	2	8	59	Para-tumor brain tissue	-	Wild-type	0.024078908	Wild-type
27	Male	12	Pilocyticastrocytoma	1	1	60	Tumor tissue	-	Wild-type	0.025381608	Wild-type
28	Female	39	Pilocyticastrocytoma	1	1	61	Tumor tissue	-	Wild-type	0.060065671	Wild-type
29	Male	60	Glioblastoma	4	1	62	Tumor tissue	-+	Wild-type	0.00839673	Wild-type
30	Male	43	Astrocytoma	3	1	63	Tumor tissue	+	R132H	4.071231176	IDH mutation
31	Male	48	Glioblastoma	4	1	64	Tumor tissue	-	Wild-type	0.042984702	Wild-type
32	Male	43	Astrocytoma	3	1	65	Tumor tissue	+	R132H	0.915080404	IDH mutation
	Male	43	Astrocytoma	3	2	66	Tumor tissue	+	R132H	1.03460014	IDH mutation
33	Male	66	Glioblastoma	4	1	67	Tumor tissue	-	Wild-type	0.070692941	Wild-type
34	Male	33	Oligodendroglioma	2	1	68	Tumor tissue	+	R132H	3.726829428	IDH mutation
35	Male	57	Glioblastoma	4	1	69	Tumor tissue	-	Wild-type	0.061558282	Wild-type
36	Female	39	Oligodendroglioma	3	1	70	Tumor tissue	+	R132H	3.096650017	IDH mutation
	Female	39	Oligodendroglioma	3	2	71	Tumor tissue	+	R132H	1.969162071	IDH mutation
37	Female	28	Glioblastoma	4	1	72	Tumor tissue	+-	Wild-type	0.040290193	Wild-type
	Female	28	Glioblastoma	4	2	73	Tumor tissue	+-	Wild-type	0.081380238	Wild-type
	Female	28	Glioblastoma	4	3	74	Tumor tissue	+-	Wild-type	0.03282032	Wild-type
	Female	28	Glioblastoma	4	4	75	Tumor tissue	+-	Wild-type	0.060762607	Wild-type
	Female	28	Glioblastoma	4	5	76	Tumor tissue	+-	Wild-type	0.024078908	Wild-type
	Female	28	Glioblastoma	4	6	77	Tumor tissue	+-	Wild-type	0.140732014	Wild-type
	Female	28	Glioblastoma	4	7	78	Tumor tissue	+-	Wild-type	0.047992829	Wild-type
38	Male	28	Glioblastoma	4	1	79	Tumor tissue	-	Wild-type	0.001692309	Wild-type
	Male	28	Glioblastoma	4	2	80	Tumor tissue	-	Wild-type	0.014027336	Wild-type
	Male	28	Glioblastoma	4	3	81	Tumor tissue	-	Wild-type	0.021424352	Wild-type
	Male	28	Glioblastoma	4	4	82	Tumor tissue	-	Wild-type	0.00893329	Wild-type

39	Female	56	Astrocytoma	3	1	83	Tumor tissue	+	R132H	6.032216577	IDH mutation
	Female	56	Astrocytoma	3	2	84	Tumor tissue	+	R132H	11.26631489	IDH mutation
	Female	56	Astrocytoma	3	3	85	Tumor tissue	+	R132H	5.647824224	IDH mutation
	Female	56	Astrocytoma	3	4	86	Tumor tissue	+	R132H	0.626821919	IDH mutation
	Female	56	Astrocytoma	3	5	87	Tumor tissue	+	R132H	0.637470487	IDH mutation
	Female	56	Astrocytoma	3	6	88	Tumor tissue	+	R132H	0.595347225	IDH mutation
	Female	56	Astrocytoma	3	7	89	Tumor tissue	+	R132H	0.621224407	IDH mutation
	Female	56	Astrocytoma	3	8	90	Para-tumor brain tissue	-	Wild-type	0.076131332	Wild-type
	Female	56	Astrocytoma	3	9	91	Para-tumor brain tissue	-	Wild-type	0.037251999	Wild-type
	Female	56	Astrocytoma	3	10	92	Para-tumor brain tissue	-	Wild-type	0.132251287	Wild-type
40	Male	42	Astrocytoma	2	1	93	Tumor tissue	+	R132H	8.23183173	IDH mutation
41	Male	44	Glioblastoma	4	1	94	Tumor tissue	-	Wild-type	0.046183192	Wild-type
	Male	44	Glioblastoma	4	2	95	Tumor tissue	-	Wild-type	0.032733976	Wild-type
	Male	44	Glioblastoma	4	3	96	Tumor tissue	-	Wild-type	0.011264025	Wild-type
	Male	44	Glioblastoma	4	4	97	Tumor tissue	-	Wild-type	0.021528895	Wild-type
	Male	44	Glioblastoma	4	5	98	Tumor tissue	-	Wild-type	0.000145381	Wild-type
	Male	44	Glioblastoma	4	6	99	Tumor tissue	-	Wild-type	0.117740811	Wild-type
42	Female	74	Glioblastoma	4	1	100	Tumor tissue	-	Wild-type	0.142843296	Wild-type
43	Female	19	Pilocyticastrocytoma	1	1	101	Tumor tissue	-	Wild-type	0.04796373	Wild-type
44	Male	69	Glioblastoma	4	1	102	Tumor tissue	-	Wild-type	0.035895901	Wild-type
45	Male	33	Glioblastoma	4	1	103	Tumor tissue	-	Wild-type	0.071553168	Wild-type
	Male	33	Glioblastoma	4	2	104	Tumor tissue	-	Wild-type	0.022378408	Wild-type
46	Male	61	Glioblastoma	4	1	105	Tumor tissue	-	Wild-type	0.061318592	Wild-type
47	Male	74	Glioblastoma	4	1	106	Tumor tissue	-	Wild-type	0.072462325	Wild-type
48	Female	36	Oligodendroglioma	2	1	107	Tumor tissue	+	R132H	0.24369536	IDH mutation
49	Female	57	Glioblastoma	4	1	108	Tumor tissue	-	Wild-type	0.051249322	Wild-type
50	Male	57	Astrocytoma	4	1	109	Tumor tissue	-	Wild-type	0.030852779	Wild-type
	Male	57	Astrocytoma	4	2	110	Tumor tissue	-	Wild-type	3.27787E-05	Wild-type
	Male	57	Astrocytoma	4	3	111	Tumor tissue	-	Wild-type	0.047221446	Wild-type

51	Male	57	Astrocytoma	4	4	112	Tumor tissue	-	Wild-type	0.045817845	Wild-type
	Male	66	Glioblastoma	4	1	113	Tumor tissue	-	Wild-type	0.078321058	Wild-type
	Male	66	Glioblastoma	4	2	114	Tumor tissue	-	Wild-type	0.019953554	Wild-type
52	Female	53	Astrocytoma	2	1	115	Tumor tissue	-	Wild-type	0.024472014	Wild-type
	Female	53	Astrocytoma	2	2	116	Tumor tissue	-	Wild-type	0.051855861	Wild-type
	Female	53	Astrocytoma	2	3	117	Tumor tissue	-	Wild-type	0.04747865	Wild-type
53	Male	32	Oligodendroglioma	3	1	118	Tumor tissue	+	R132H	1.946516608	IDH mutation
54	Male	43	Astrocytoma	2	1	119	Tumor tissue	+	R132H	1.999103748	IDH mutation
55	Male	21	Glioblastoma	4	1	120	Tumor tissue	-	Wild-type	0.084484347	Wild-type
56	Female	65	Glioblastoma	4	1	121	Tumor tissue	-	Wild-type	0.04288887	Wild-type
	Female	65	Glioblastoma	4	2	122	Tumor tissue	-	Wild-type	0.011677865	Wild-type
	Female	65	Glioblastoma	4	3	123	Tumor tissue	-	Wild-type	0.009328605	Wild-type
	Female	65	Glioblastoma	4	4	124	Tumor tissue	-	Wild-type	0.028352712	Wild-type
	Female	65	Glioblastoma	4	5	125	Tumor tissue	-	Wild-type	0.051923635	Wild-type
	Female	65	Glioblastoma	4	6	126	Tumor tissue	-	Wild-type	0.035165654	Wild-type
	Female	65	Glioblastoma	4	7	127	Tumor tissue	-	Wild-type	0.045838608	Wild-type
57	Female	44	Oligodendroglioma	2	1	128	Tumor tissue	+	R132H	0.516849534	IDH mutation
	Female	44	Oligodendroglioma	2	2	129	Tumor tissue	+	R132H	0.47209414	IDH mutation
58	Male	67	Glioblastoma	4	1	130	Tumor tissue	-	Wild-type	0.001812215	Wild-type
59	Male	57	Astrocytoma	2	1	131	Tumor tissue	-	Wild-type	0.057542496	Wild-type
60	Male	58	Glioblastoma	4	1	132	Tumor tissue	-	Wild-type	0.03489292	Wild-type
61	Male	42	Oligodendroglioma	2	1	133	Tumor tissue	+	R132H	0.446777706	IDH mutation
	Male	42	Oligodendroglioma	2	2	134	Tumor tissue	+	R132H	0.552756104	IDH mutation
	Male	42	Oligodendroglioma	2	3	135	Tumor tissue	+	R132H	0.806040716	IDH mutation
	Male	42	Oligodendroglioma	2	4	136	Tumor tissue	+	R132H	0.950223138	IDH mutation
	Male	42	Oligodendroglioma	2	5	137	Tumor tissue	+	R132H	1.025521335	IDH mutation
	Male	42	Oligodendroglioma	2	6	138	Tumor tissue	+	R132H	1.139042364	IDH mutation
	Male	42	Oligodendroglioma	2	7	139	Tumor tissue	+	R132H	1.903361423	IDH mutation
	Male	42	Oligodendroglioma	2	8	140	Tumor tissue	+	R132H	2.003727206	IDH mutation

	Male	42	Oligodendroglioma	2	9	141	Tumor tissue	+	R132H	2.41629913	IDH mutation
	Male	42	Oligodendroglioma	2	10	142	Tumor tissue	+	R132H	3.056662809	IDH mutation
	Male	42	Oligodendroglioma	2	11	143	Tumor tissue	+	R132H	3.738562893	IDH mutation
	Male	42	Oligodendroglioma	2	12	144	Tumor tissue	+	R132H	7.651925794	IDH mutation
	Male	42	Oligodendroglioma	2	13	145	Para-tumor brain tissue	-	Wild-type	0.019913518	Wild-type
	Male	42	Oligodendroglioma	2	14	146	Para-tumor brain tissue	-	Wild-type	0.029994431	Wild-type
	Male	42	Oligodendroglioma	2	15	147	Para-tumor brain tissue	-	Wild-type	0.020008911	Wild-type
62	Male	41	Oligodendroglioma	2	1	148	Tumor tissue	+	R132H	0.692355761	IDH mutation
	Male	41	Oligodendroglioma	2	2	149	Tumor tissue	+	R132H	0.869160106	IDH mutation
63	Male	44	Glioblastoma	4	1	150	Tumor tissue	-	Wild-type	0.04905914	Wild-type
	Male	44	Glioblastoma	4	2	151	Tumor tissue	-	Wild-type	0.023605598	Wild-type
64	Female	49	Astrocytoma	2	1	152	Tumor tissue	+	R132H	0.481862051	IDH mutation
	Male	31	Astrocytoma	2	1	153	Tumor tissue	+	R132H	8.141702703	IDH mutation
	Male	31	Astrocytoma	2	2	154	Tumor tissue	+	R132H	1.346643312	IDH mutation
	Male	31	Astrocytoma	2	3	155	Tumor tissue	+	R132H	2.886295423	IDH mutation
	Male	31	Astrocytoma	2	4	156	Tumor tissue	+	R132H	1.805250557	IDH mutation
65	Male	31	Astrocytoma	2	5	157	Tumor tissue	+	R132H	0.49845122	IDH mutation
	Male	31	Astrocytoma	2	6	158	Tumor tissue	+	R132H	9.972707865	IDH mutation
	Male	31	Astrocytoma	2	7	159	Tumor tissue	+	R132H	1.804707434	IDH mutation
	Male	31	Astrocytoma	2	8	160	Tumor tissue	+	R132H	3.520632653	IDH mutation
	Male	31	Astrocytoma	2	9	161	Para-tumor brain tissue	-	Wild-type	0.009576541	Wild-type
66	Female	56	Glioblastoma	4	1	162	Tumor tissue	-	Wild-type	0.074435993	Wild-type
	Female	56	Glioblastoma	4	2	163	Tumor tissue	-	Wild-type	0.052135663	Wild-type
67	Female	40	Astrocytoma	2	1	164	Tumor tissue	+	R132H	1.790919561	IDH mutation
	Female	40	Astrocytoma	2	2	165	Tumor tissue	+	R132H	1.706885533	IDH mutation
68	Male	31	Oligodendroglioma	2	1	166	Tumor tissue	+	R132H	6.816419355	IDH mutation
69	Male	54	Glioblastoma	4	1	167	Tumor tissue	-	Wild-type	0.164721785	Wild-type
70	Male	63	Glioblastoma	4	1	168	Tumor tissue	-	Wild-type	0.043765123	Wild-type
71	Female	29	Neurilemmoma	1	1	169	Normal brain tissue	-	Wild-type	0.04203219	Wild-type

72	Male	46	Meningioma	1	1	170	Normal brain tissue	-	Wild-type	0.001512389	Wild-type
73	Male	70	Meningioma	1	1	171	Normal brain tissue	-	Wild-type	0.054875757	Wild-type
74	Female	47	Meningioma	1	1	172	Normal brain tissue	-	Wild-type	0.038171751	Wild-type

Video showing tissue collection and analysis by DESI-MS to illustrate the speed of mutation status determination

A video showing the standard of care for the treatment of a patient suspected of glioma tumor undergoing craniotomy surgery. The sequential steps of the surgical resection are demonstrated. The freshly resected tissue samples from different locations of the tumor are transferred to the DESI-MS operator, and then, a small piece of each tissue is smeared on a glass slide by a 3D-printed smearer tool. The biopsy smear is placed on the DESI source for performing tandem MS measurements. The fragment ions of 2HG and Glu are simultaneously detected and measured in MS/MS mode. IDH-mut tumors show significantly higher 2HG signals compared to Glu.

References

1. J. S. Rossi, "Statistical Power Analysis" in *Handbook of Psychology, Second Edition*, (2012)
2. Z. Takáts, J. M. Wiseman, R. G. Cooks, Ambient mass spectrometry using desorption electrospray ionization (DESI): instrumentation, mechanisms and applications in forensics, chemistry, and biology. *Journal of Mass Spectrometry* **40**, 1261–1275 (2005).
3. C. M. Alfaro, *et al.*, Intraoperative assessment of isocitrate dehydrogenase mutation status in human gliomas using desorption electrospray ionization–mass spectrometry. *JNS* **132**, 180–187 (2020).
4. V. Pirro, *et al.*, Intraoperative assessment of tumor margins during glioma resection by desorption electrospray ionization-mass spectrometry. *Proc. Natl. Acad. Sci. U. S.* **114**, 6700–6705 (2017).
5. H. M. Brown, *et al.*, Intraoperative Mass Spectrometry Platform for IDH Mutation Status Prediction, Glioma Diagnosis, and Estimation of Tumor Cell Infiltration. *J Appl Lab Med* **6**, 902–916 (2021).
6. Y. Ren, M. N. McLuckey, J. Liu, Z. Ouyang, Direct mass spectrometry analysis of biofluid samples using slug-flow microextraction nano-electrospray ionization. *Angew. Chem., Int. Ed. Engl.* **53**, 14124–14127 (2014).
7. R. Zou, *et al.*, Point-of-Care Tissue Analysis Using Miniature Mass Spectrometer. *Anal. Chem.* **91**, 1157–1163 (2019).
8. S. K. McBrayer, *et al.*, Transaminase Inhibition by 2-Hydroxyglutarate Impairs Glutamate Biosynthesis and Redox Homeostasis in Glioma. *Cell* **175**, 101-116.e25 (2018).
9. H. M. Brown, *et al.*, Intraoperative detection of isocitrate dehydrogenase mutations in human gliomas using a miniature mass spectrometer. *Anal. Bioanal. Chem.* **411**, 7929–7933 (2019).
